# An Open‐Source Pipeline for Calcium Imaging and All‐Optical Physiology in Human Stem Cell‐Derived Neurons

**DOI:** 10.1002/advs.202515887

**Published:** 2026-03-09

**Authors:** Wardiya Afshar‐Saber, Federico M. Gasparoli, Ziqin Yang, Nicole A. Teaney, Rachel Hobson, Lahin Lalani, Gayathri Srinivasan, Dosh Whye, Ranit Karmakar, Elizabeth D. Buttermore, Kellen D. Winden, Cidi Chen, Mustafa Sahin

**Affiliations:** ^1^ Department of Neurology, F.M. Kirby Neurobiology Center, Harvard Medical School Boston Children's Hospital Boston Massachusetts USA; ^2^ Rosamund Stone Zander and Hansjoerg Wyss Translational Neuroscience Center Boston Massachusetts USA; ^3^ Department of Systems Biology Harvard Medical School Boston Massachusetts USA; ^4^ Human Neuron Core Boston Children's Hospital Boston Massachusetts USA

**Keywords:** calcium imaging, machine learning, neurodevelopmental disorders, open‐source, optogenetics, stem cells

## Abstract

High‐throughput, single‐cell resolution profiling of neuronal activity is critical for understanding brain function and modeling neurological disorders, yet existing approaches are often limited by scalability and manual workflows. Here, we present an open‐source, scalable imaging and analysis platform that integrates optogenetic stimulation, calcium imaging, automated acquisition, single‐cell and network analyses. The platform enables robust quantification of spontaneous and evoked neuronal activity across hundreds of human stem cell‐derived neurons over multiple timepoints, supporting functional phenotyping at both cellular and network levels. We demonstrate the versatility of the platform across multiple disease‐relevant contexts, including models of CDKL5 Deficiency, SSADH Deficiency, and tuberous sclerosis complex (TSC). Additionally, we generate CRISPR‐Cas9 knock‐in hiPSC lines expressing GCaMP6s and demonstrate partial reversal through pharmacological intervention in TSC. By linking single‐cell dynamics to network‐level measures, this platform provides a generalizable framework for scalable functional phenotyping and high‐throughput screening in human neuronal models.

## Graphical abstract

This work introduces an open‐source all‐optical platform for functional phenotyping of human stem cell‐derived neurons. The system integrates optogenetics, calcium imaging, automated acquisition, and analysis to resolve single‐cell and network activity, enabling longitudinal measurements, disease modeling, and pharmacological screening in preclinical models of neurodevelopmental disorders.

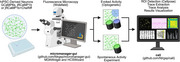

## 1 Introduction

High‐throughput, single‐cell resolution profiling of neuronal activity is essential for elucidating the mechanisms underlying circuit function, modeling human neurological diseases, and developing targeted therapeutics [1, 2]. Traditional electrophysiological techniques such as patch‐clamp recording and multi‐electrode arrays (MEAs) remain gold standards for functional interrogation of neuronal networks, but their limited scalability, throughput, and spatial resolution constrain their application for large‐scale studies of heterogeneous neural populations, particularly in patient‐derived stem cell models, including multi‐well conditions, and multiple timepoints [2, 3, 4]. Genetically encoded calcium indicators (GECIs) have transformed functional imaging of neuronal activity, enabling non‐invasive recordings of large neuronal populations with single‐cell resolution [5, 6]. When combined with optogenetic actuators [7], these technologies enable all‐optical approaches for dissecting network connectivity and synaptic function offering unparalleled opportunities for studying neural circuit dynamics in both health and disease [8, 9].

Several neurodevelopmental disorders, such as tuberous sclerosis complex (TSC), CDKL5 Deficiency Disorder (CDD), and Succinic Semialdehyde Dehydrogenase Deficiency (SSADHD) are characterized by alterations in excitability and synchronization that contribute to the pathogenesis of epilepsy [10, 11, 12, 13]. Human induced pluripotent stem cell (hiPSC)‐derived neuronal models provide a unique opportunity to dissect the altered neuronal activity in human neurons [14, 15, 16]; however, scalable tools for quantifying network and single‐cell level activities across large populations remain limited. Additionally, most applications of GECIs in human stem cell‐derived neuronal models still rely on viral transduction of postmitotic neurons.

While viral delivery is convenient and versatile, it requires costly, large‐scale production of viral particles and can introduce toxicity as well as variability in transduction efficiency across biological and technical replicates [17, 18]. In contrast, targeted stable genomic integration of GECIs into safe‐harbor loci via genome editing enables earlier expression during neural differentiation, allowing activity to be recorded from the earliest stages of network formation. This approach supports longitudinal imaging of circuit maturation and chronic pharmacological interventions while also ensuring consistency across hiPSC lines and differentiation protocols [19, 20, 21].

Beyond the technical aspects of indicator expression, the lack of robust, scalable acquisition and analysis pipelines remains a major barrier to the routine implementation of all‐optical functional assays in stem cell‐derived neuronal models. Calcium imaging datasets pose distinct challenges for reliable cell segmentation, extraction of single‐cell activity traces, and quantification of network‐level dynamics [22]. Although several software tools offer powerful analysis features, they are often limited by their reliance on manual or semi‐automated segmentation [23, 24], are optimized for in vivo applications with the lack of essential features such as plate mapping [22] or limited adaptability to in vitro all‐optical physiology [25]. Fully automated acquisition and analysis pipelines that reduce manual input while enabling high‐content, single‐cell resolution are essential to realizing the full potential of these approaches for disease modeling and drug discovery.

**FIGURE 1:**
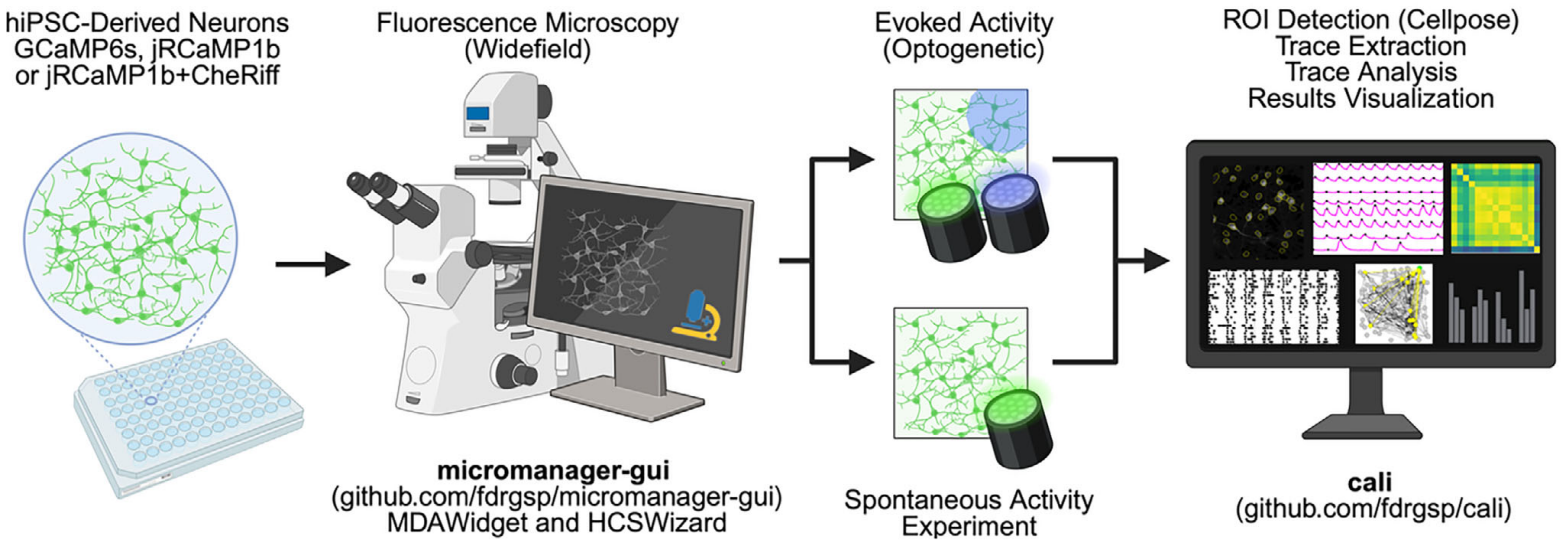
Workflow. Human iPSC–derived neuronal cultures expressing genetically encoded calcium indicators (GCaMP6s or jRCaMP1b) are plated in multi‐well plates and imaged on a widefield microscope controlled by the open‐source, Python‐based micromanager‐gui software (https://github.com/fdrgsp/micromanager‐gui). After a simple plate calibration, micromanager‐gui enables fully automated, reproducible multi‐well time‐lapse acquisitions with unbiased field‐of‐view selection in each well. The same system can record spontaneous network activity or, using a cost‐effective optogenetic illumination path, deliver spatially and temporally light stimulation across the entire field of view or targeted sub‐regions (in our work using jRCaMP1b with CheRiff). Acquired image sequences can be opened directly in the open‐source, Python‐based *cali* package (https://github.com/fdrgsp/cali), which performs ROI detection, calcium trace extraction, quantitative analysis, and interactive visualization. Created in BioRender. Saber, W. (2026) https://BioRender.com/68iwjcp.

Here, we present a fully integrated experimental and analytical framework for scalable, high‐content calcium imaging capable of extracting multidimensional functional features at a single‐cell level from human hiPSC‐derived neuronal models (Figure 1). In addition to the commonly used viral transduction method, we knocked in GCaMP6s at the AAVS1 safe‐harbor locus using CRISPR‐Cas9 and generated hiPSC lines with stable GECI expression. This is combined with a modular, open‐source acquisition platform enabling optogenetic stimulation, deep learning‐based cell segmentation, and single‐cell calcium dynamics quantification via the *cali* analysis interface. We demonstrated the potential of this approach across multiple differentiation methods and neurodevelopmental models, including TSC, CDD, and SSADHD, which share epilepsy as a core clinical feature. We further validated its suitability for pharmacological screening by applying potassium channel modulators to normalize hyperexcitability in *TSC2*‐deficient neurons. This integrated platform provides a scalable, reproducible solution for high‐throughput functional screening and mechanistic investigation of circuit‐level phenotypes in human disease models, with broad applicability for both basic neuroscience and therapeutic development.

## 2 Results

### 2.1 Generation of Stem Cell‐Derived Human Neurons for Calcium Imaging and All‐Optical Assay

**FIGURE 2:**
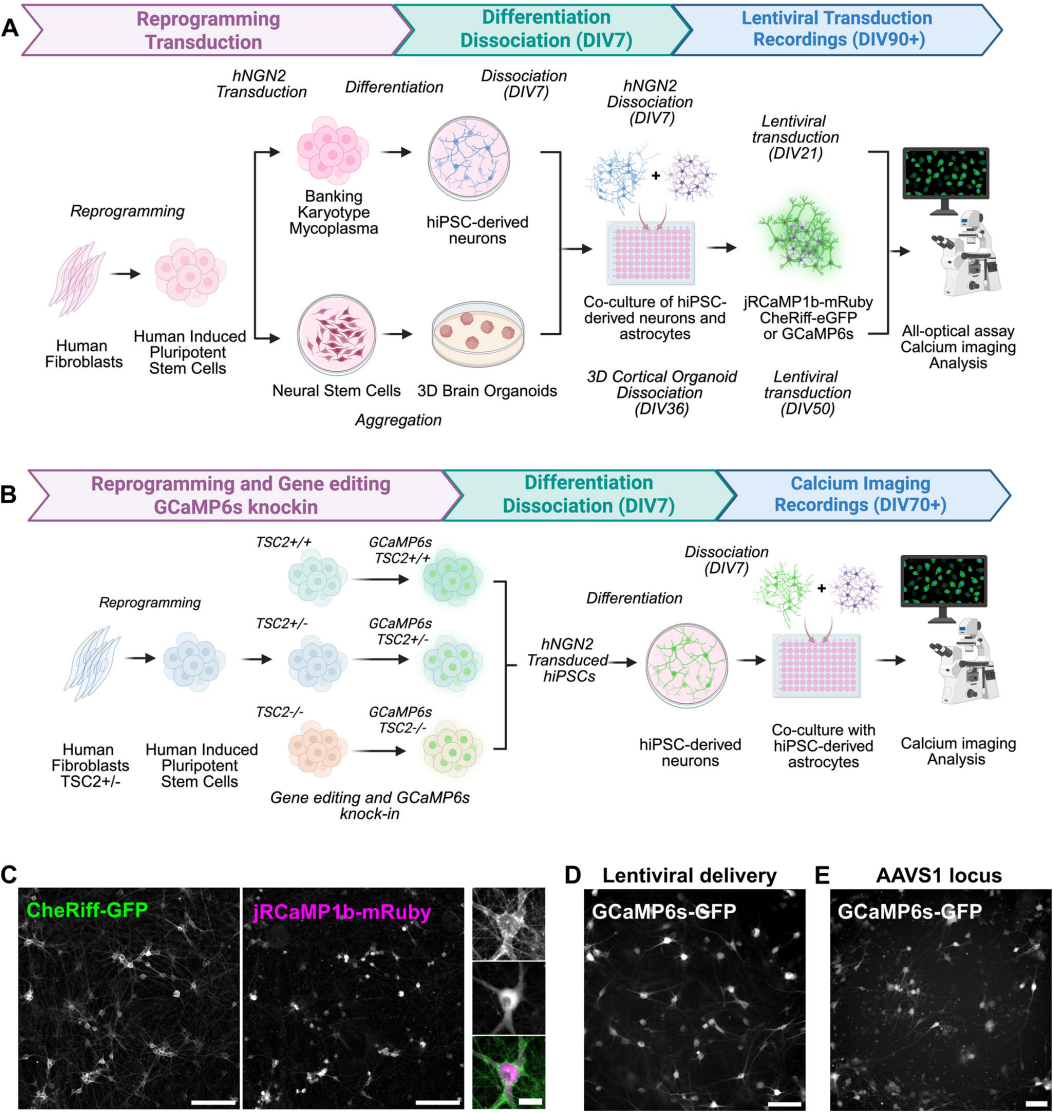
Generation of stem cell‐derived neurons for calcium imaging and all‐optical physiology. (A) Experimental timeline starting with the reprogramming of human fibroblasts into hiPSCs, followed either by transduction with hNGN2, differentiation and co‐culture with hiPSC‐derived astrocytes or generation of neural stem cells, 3D cortical organoids followed by dissociation and co‐culture with hiPSC‐derived astrocytes. Transduction of the calcium indicators for calcium imaging or all‐optical assay. (B) Experimental timeline starting with the reprograming of human fibroblasts from TSC patients into hiPSCs, followed by gene editing to generate the full allelic series and CRISPR‐mediated GCaMP6s knock‐in in the safe harbor locus. After banking, transduction with hNGN2, differentiation and co‐culture with hiPSC‐derived astrocytes for calcium imaging. Created in BioRender. Saber, W. (2026) https://BioRender.com/1is3s0w. (C) Expression of CheRiff‐GFP and jRCaMP1b‐mRuby in a co‐culture of hiPSC‐derived neurons and astrocytes at DIV 30, scale bars 200 and 30 µm. (D,E) Expression of GCaMP6s‐GFP in a co‐culture of hiPSC‐derived neurons and astrocytes at DIV 30 following lentiviral transduction (D), or safe harbor locus method (E), scale bar 100 µm.

We generated excitatory neurons using two approaches. On one hand, we transduced hiPSCs with a lentiviral vector encoding human NGN2 (hNGN2) under a tetracycline‐inducible promoter, alongside a puromycin resistance gene for selection and enrichment (Figure 2A,B) [26]. On the other hand, we formed and dissociated 3D cortical organoids differentiated from hiPSCs at 36 days in vitro (DIV) (Figure 2A) [27]. To support neuronal maturation of both types of differentiating neurons, we co‐cultured these with hiPSC‐derived astrocytes for ∼50 days. On DIV21 of the hNGN2 differentiation and DIV50 of the organoid‐based differentiation, we transduced the co‐culture of neurons and astrocytes with lentiviral constructs encoding either the GFP‐based calcium indicator GCaMP6s or the red‐shifted calcium indicator jRCaMP1b and the optogenetic actuator CheRiff, both driven by the human synapsin promoter (pLV‐hSynGCaMP6s, pLV‐hSyn‐CheRiff‐eGFP and pLV‐hSyn‐jRCaMP1b‐mRuby) (Figure 2A). One week post‐transduction, we observed robust, consistent expression of GFP and mRuby using both differentiation methods, confirming successful transduction of these reporters (Figure 2C,D).

While viral transduction is commonly employed to deliver GECIs into postmitotic neurons, this approach can introduce variability between differentiation batches, posing a challenge for reproducibility in large‐scale studies, including multi‐well, conditions, and multiple timepoints [18]. To overcome these limitations, we also constitutively expressed the GECI *GCaMP6s*‐GFP in the AAVS1 safe harbor locus of three hiPSCs lines via CRISPR‐Cas9 homologous recombination (Figure 2B, Figure S1A) [19]. The first line was derived from a patient with TSC due to an 18 bp in‐frame deletion in exon 40 of *TSC2* (c.5238_5255del p.H1746_R1751del, *TSC2* ^+/−^). The second line was derived from the patient line and had a frameshift mutation induced in the second allele of *TSC2* using TALEN technology (*TSC2* ^−/−^), modeling loss‐of‐function that is observed in TSC. Finally, we generated a third isogenic control hiPSC line (*TSC2* ^+/+^) using CRISPR‐Cas9 (Table 1) [28]. We confirmed successful knock‐in clones with the GCaMP6s expression cassette at the AAVS1 (Figure S1B,C), verified the maintenance of pluripotency by analyzing the expression of pluripotency markers SSEA4, OCT4, Nanog, and TRA‐1–60, and did not detect any karyotypic abnormalities (Figure S1D,E). Finally, the detection of GFP signal in differentiated neurons demonstrated both robust GCaMP6s expression and accurate integration into the safe harbor locus (Figure 2E, Figure S1F).

**TABLE 1:** Summary table of the hiPSC lines used in this study.

Disorder	Line name	Sex	Genotype
Tuberous Sclerosis Complex (TSC)	77 line‐*TSC2* ^+/−^	F	c.5238_5255del p.H1746_R1751del (heterozygous)
	77 line‐*TSC2* ^−/−^	F	TALEN‐LOF: *TSC2* ^−/−^ (homozygous)
	77 line‐*TSC2* ^+/+^	F	CRISPR‐Cas9: *TSC2* ^+/+^ (homozygous)
CDKL5 Deficiency (CDD)	HNDS0083‐01#B (BCHi005‐A)	F	c.1648C>T, p.Arg550* (heterozygous)
	Sex matched parental control	F	c.1648C (homozygous)
SSADH Deficiency (SSADHD)	HNDS0005‐01#B (BCHi007‐A)	F	*ALDH5A1* ^−/−^c.1226G > A / c.1226G > A; p.Gly409Asp
	Sex matched parental control	F	*ALDH5A1* ^+/−^ c.1226G / c.1226G > A; p.Gly409Asp

### 2.2 Modular Acquisition Platform for Calcium Imaging and Optogenetics

**FIGURE 3:**
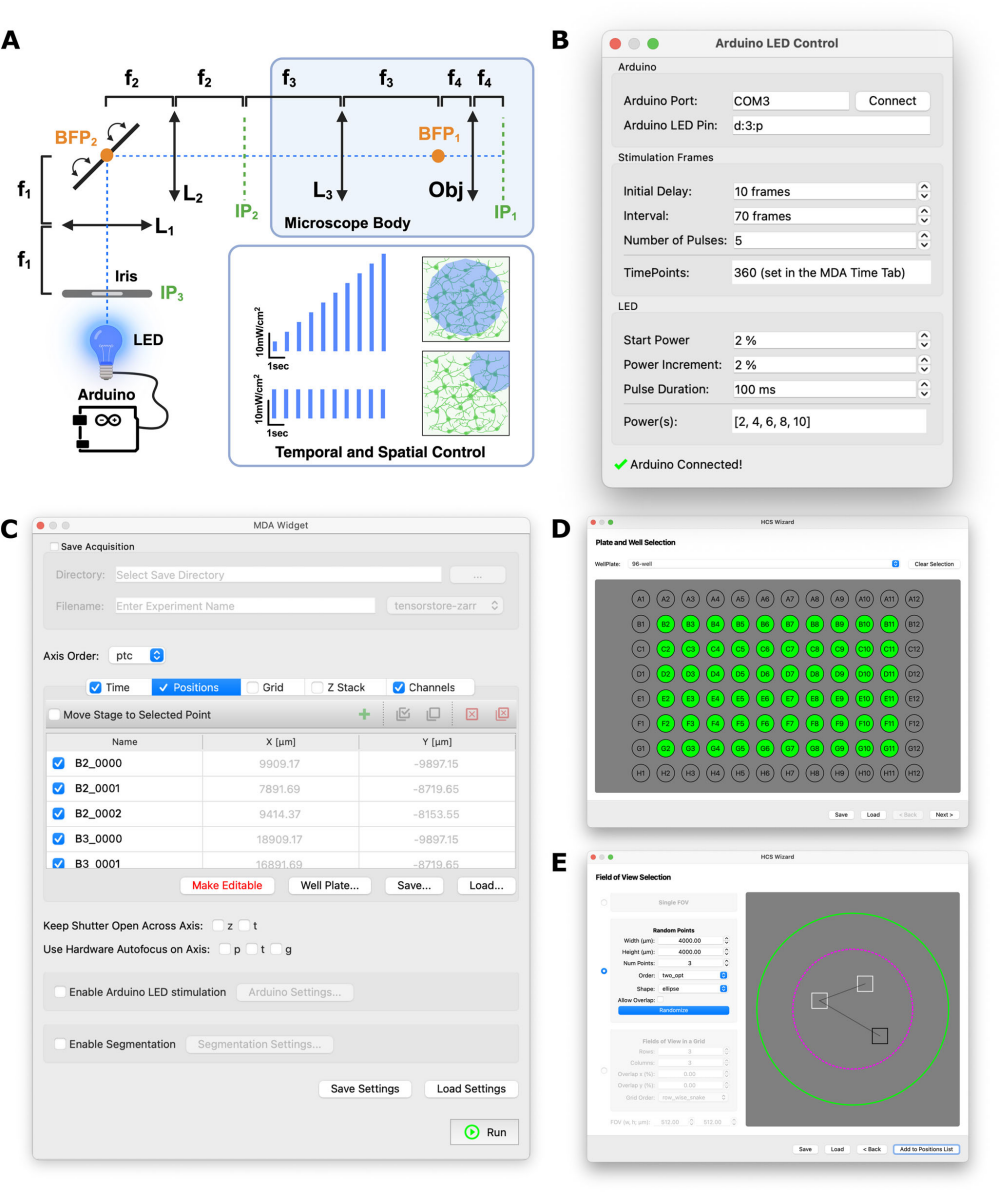
Modular acquisition platform for calcium imaging and all‐optical physiology. (A) Optical path for evoked activity experiment. A manual lever‐actuated iris diaphragm is installed after a blue LED in a plane conjugated with the focal plane of the microscope objective. In this way, within the field of view, different sizes of the blue illumination could be selected. An elliptical mirror mounted on a manual kinematic mount is placed in a plane conjugated with the back focal plane of the objective so that steering it would allow for positioning the blue illumination in different parts of the FOV. Created in BioRender. Saber, W. (2026) https://BioRender.com/7j5z2of. (B) Arduino‐based control module for LED pulsing, enabling programmable control of pulse timing, duration, and intensity. (C–E) micromanager‐gui modules: (C) the MDAWidget to setup multi‐dimensional acquisition experiments (D,E) and the MDAWizard to configure multi‐well plate acquisitions (wells and FOVs).

We developed an open‐source acquisition platform incorporating three key features: (1) a customizable multi‐dimensional acquisition (MDA) interface, (2) an optogenetic stimulation control, and (3) a Slackbot‐based remote acquisition control (Figure 3). This platform allowed us to perform large‐scale, automated imaging experiments across multi‐well plates with high reproducibility and minimal manual intervention (Figure S2 and Video S1).

We built our platform on the Micro‐Manager ecosystem [29], leveraging its broad hardware compatibility. However, limitations in Micro‐Manager's Java‐based architecture made it difficult to support synchronized stimulation, real‐time analysis, and Python‐based workflows. To address this, we developed a modular graphical user interface (GUI) using the pymmcore‐plus library, which provides a Python‐native interface to the Micro‐Manager C++ core. This allowed for rapid customization, seamless integration with modern analysis tools, and direct control of acquisition workflows. Using pymmcore‐plus, along with pymmcore‐widgets and useq‐schema (https://pymmcore‐plus.github.io/pymmcore‐plus), we developed micromanager‐gui (https://github.com/fdrgsp/micromanager‐gui), an extensible GUI tailored for calcium imaging and optogenetics experiments (Figure S2). The software remains fully compatible with standard Micro‐Manager configuration files, allowing users to adopt the platform without modifying existing microscope setups (Video S1).

To support precise optogenetic stimulation, we developed a dedicated Arduino‐based control module for LED pulsing, enabling programmable control of pulse timing, duration, and intensity (Figure 3A,B) [8]. Spatial targeting was achieved using a diaphragm at the sample plane and a kinematically mounted mirror at the back focal plane, allowing us to restrict stimulation to specific subregions of the imaging field (e.g., one corner of a well; Figure 3A).

Multi‐dimensional acquisition (MDA) experiments can be configured using the *MDAWidget*, which supports standard imaging modes including multi‐channel, time‐lapse, Z‐stack, multi‐position, and grid acquisitions (Figure 3C, Figure S2). The widget allows acquisition settings to be saved and reloaded, ensuring reproducibility across experiments and users. Integration with hardware autofocus (Nikon PFS) maintained stable focus across wells during long‐term imaging sessions. To facilitate unbiased and reproducible high‐content imaging, we incorporated the *HCSWizard*, a widget that enables multi‐well plates calibration and flexible field‐of‐view (FOV) selection across standard formats (e.g., 96‐ or 384‐well plates) (Figure 3D, Figure S2 and Video S2). Users could select center‐of‐well, reproducible random, or grid‐based FOVs, providing flexibility to adapt acquisition strategies to different experimental needs while maintaining consistency and reducing selection bias (Figure 3E). Additionally, we adopted OME‐Zarr as the standard output format, leveraging Google TensorStore for asynchronous, chunked data writing during acquisition. This allowed us to capture large time‐series datasets efficiently, without memory constraints, and ensured compatibility with scalable, Python‐based analysis pipelines.

If needed, users can enable on‐the‐fly segmentation, which runs Cellpose in a separate process to detect neurons in each FOV for downstream analysis [30, 31]. Additionally, we integrated the Slack API and Slackbot functionality to enable remote control and monitoring of recording progress, enhancing both efficiency and productivity.

### 2.3 Integrated Data Exploration and Analysis via *Cali*

We developed *cali*, an interactive Python‐based graphical interface that can be directly integrated into micromanager‐gui to streamline exploration and analysis of the calcium imaging datasets acquired (Figure S3 and Video S2). *cali* enables visualization, segmentation, and quantitative analysis of multi‐well imaging experiments within an intuitive environment. Upon loading a dataset, *cali* renders an interactive plate layout, allowing users to navigate individual wells and rapidly access associated fields of view (FOVs). For each FOV, the full time‐series can be displayed, enabling direct inspection of spatiotemporal calcium dynamics at single‐field resolution. To enable single‐cell analysis, we integrated a Cellpose‐based segmentation interface capable of processing all or selected wells and FOVs [30].

**FIGURE 4:**
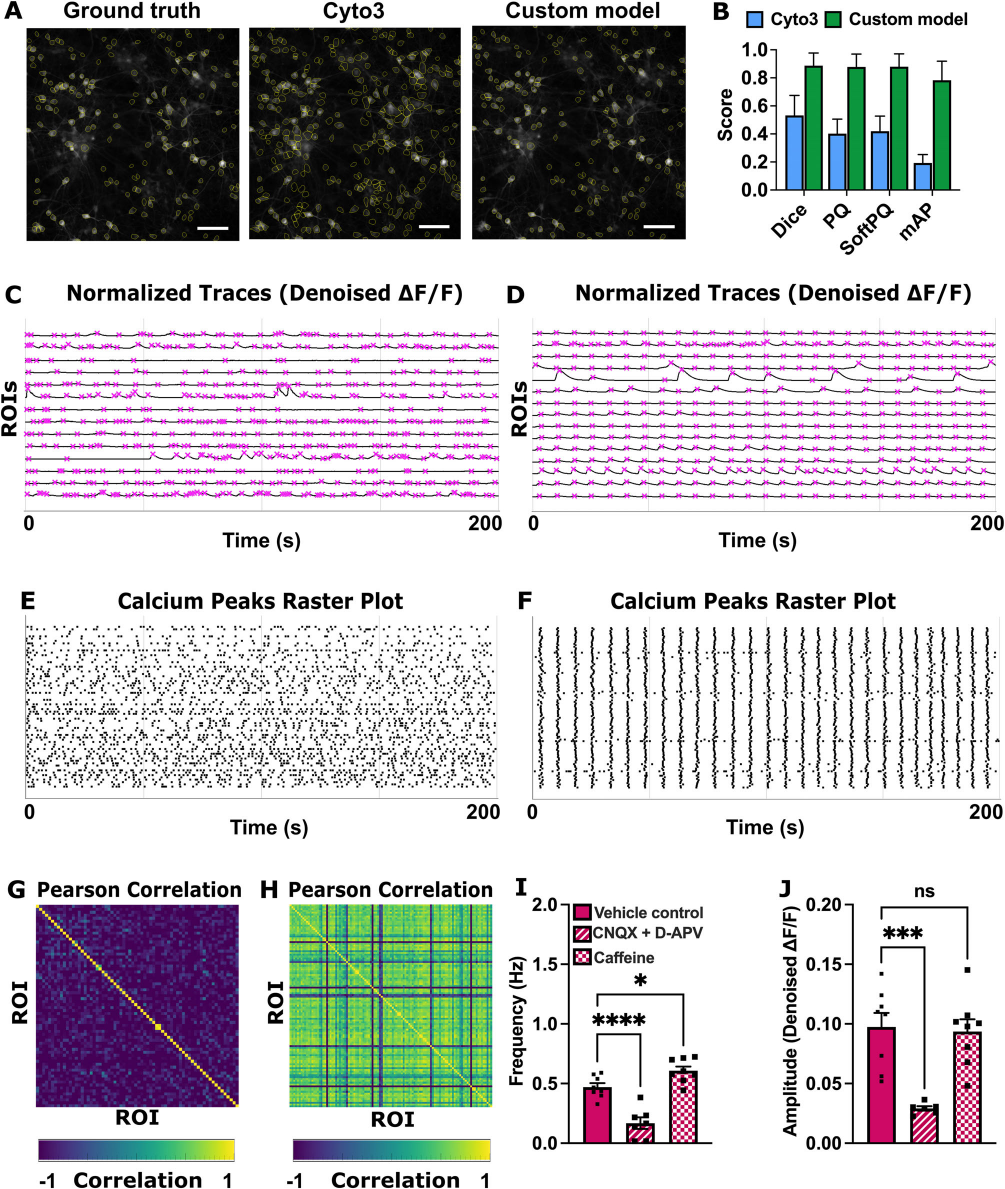
Automated segmentation and analysis pipeline for spontaneous activity. (A) Representative images of the segmentation across models. (B) Mean scores (bar height) and standard deviations (error bars) for the four segmentation metrics (SoftPQ, Dice, PQ, and mAP) calculated over the 25‐image test set. The Cellpose custom model (green) consistently achieved the highest mean scores, demonstrating superior performance and consistency compared to the generalized Cellpose Cyto3 (blue) models. (C–H) Calcium imaging analysis pipeline: (C,D) Normalized Traces (Denoised ΔF/F) of two independent FOVs and detected peaks (crosses in magenta), representative traces of 15 ROIs in the FOV are displayed. (E,F) Raster plots showing calcium transients for all individual ROIs within the FOV. (G,H) Pairwise Pearson Correlation between ROIs within the same FOV on Denoised ΔF/F Traces (G) median: 0.012 and (H) median: 0.743. (I,J) Following acute treatment with CNQX (25 µM) and D‐APV (5 µM) or caffeine (5 mM), we quantified (I) event frequency (Hz), (J) calcium event amplitude; VC: 181 neurons; CNQX and D‐APV: 161 neurons; Caffeine: 176 neurons; n = 6–8 FOVs per condition (mean ± S.E.M; ****p < 0.0001; ***p < 0.001; *p < 0.005; ns = non‐significant). One‐way ANOVA followed by Dunnett's multiple comparisons test (I) F (2, 20) = 32.81, ****p < 0.0001 and *p = 0.0342; (J) F (2, 19) = 13.46, ***p < 0.001, *p* = 0.0003.

We initially applied the built‐in Cellpose Cyto3 model and subsequently improved its segmentation accuracy by training with labeled images. We quantitatively evaluated our custom‐trained Cellpose 3 model to assess the performance improvement over the pretrained Cellpose Cyto3 model [30]. Performance was assessed on a test set of 25 images with reference instance masks (Figure 4A). For each model, we computed Dice [32], Panoptic Quality (PQ) [33], SoftPQ [34], and mean average precision (mAP) [35]. We observed a marked increase in accuracy across all metrics for the custom model. Cellpose 3 achieved Dice 0.5317, PQ 0.4025, SoftPQ 0.4198, and mAP 0.1932 (Figure 4B, Table S1). The custom Cellpose 3 model obtained substantially higher scores for all metrics: Dice 0.8869, PQ 0.8776, SoftPQ 0.8795, and mAP 0.7829 (Figure 4B, Table S1). The slight increase in SoftPQ compared to PQ accounts for a small number of undersegmentations. Furthermore, the custom Cellpose 3 model exhibited lower relative standard deviations across the test set, indicating greater robustness and consistency compared to the generalized models (Figure S4 and Table S1). Following segmentation, the resulting masks were stored alongside the dataset and served as the basis for subsequent single‐cell calcium analyses (Figure 4C–H).

In Figure 4, we displayed two representative FOVs with distinct levels of neuronal activity to demonstrate the use of *cali* (Figure 4C–H). Following segmentation, *cali* can execute a calcium imaging analysis pipeline that uses the OASIS algorithm [36] to extract raw and denoised calcium traces for each segmented neuron and, after computing ΔF/F_0_, detects calcium transients (Figure 4C,D). Detected events can be visualized as raster plots, in which each row represents an individual neuron (Figure 4E,F). *cali* can further compute different metrics including Pearson correlation matrices from denoised ΔF/F traces for each FOV which we use as a measure of population‐level connectivity. In the low‐synchrony example, the correlation matrix exhibits a low median Pearson correlation (median = 0.012; Figure 4G), whereas the high‐synchrony FOV shows a substantially higher median correlation (median = 0.743; Figure 4H), reflecting increased network‐wide correlation.

As a proof‐of‐concept, we pharmacologically modulated neuronal activity using CNQX and D‐APV, AMPA and NMDA receptor antagonists [26, 37], respectively, and caffeine, an adenosine receptor antagonist known to enhance network excitability [8, 38]. Our acquisition and analysis pipeline reliably identified ROIs across fields of view and quantified core activity features, including peak amplitude and event frequency in response to these perturbations (Figure 4I,J). Caffeine treatment significantly increased calcium event frequency compared to baseline, consistent with enhanced network excitability (Figure 4I). In contrast, synaptic blockade with CNQX/D‐APV led to a marked reduction in event frequency and a significant decrease in event amplitude, consistent with effective suppression of excitatory synaptic transmission (Figure 4I,J). Together, these results demonstrate the sensitivity and robustness of our pipeline for detecting both increases and decreases in neuronal network activity in response to pharmacological manipulation.

### 2.4 Quantification of Optically Evoked Activity

**FIGURE 5:**
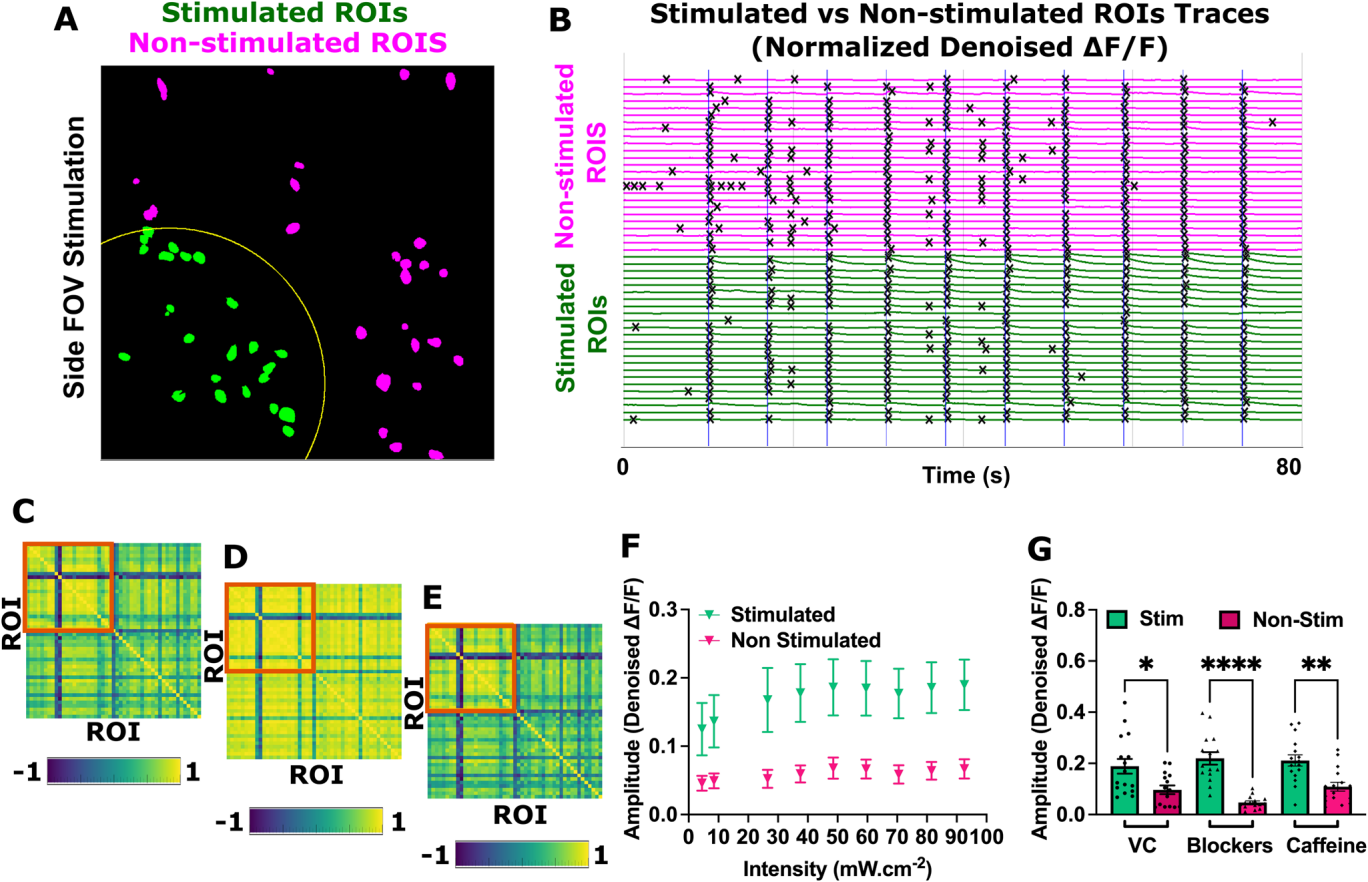
Evoked Activity Analysis Pipeline. (A) Segmentation mask using our custom model showing stimulated neurons in green within the yellow stimulation boundary; non‐stimulated cells in magenta. (B) Normalized Denoised ΔF/F from stimulated and non‐stimulated ROIs with blue lines indicating stimulation pulses. (C–E) Pearson correlation heatmaps quantify functional coupling between stimulated neurons located within the orange box and non‐stimulated neurons. Heatmap (C) shows correlations computed over the full recording, whereas heatmaps (D) and (E) depict correlations calculated specifically during stimulation (±250 ms around the stimulation event) and non‐stimulation epochs, respectively. (C) Pairwise Pearson Correlation (Denoised ΔF/F) (Sorted: 24 Stim, 25 Non‐Stim) | Stim median: 0.879 | Non‐stim median: 0.736 | Global median: 0.775 (D) Pairwise Pearson Correlation (Stim Windows ±250 ms—Denoised ΔF/F) (Sorted: 24 Stim, 25 Non‐Stim) | Stim median: 0.959 | Non‐stim median: 0.905 | Global median: 0.923 (E) Pairwise Pearson Correlation (Non‐Stim Periods, Excluding ±250 ms—Denoised ΔF/F) (Sorted: 24 Stim, 25 Non‐Stim) | Stim median: 0.863 | Non‐stim median: 0.665 | Global median: 0.730. (F) Calcium event amplitudes in stimulated (green) vs. non‐stimulated (magenta) neurons across increasing light intensities. (G) Calcium event amplitudes in stimulated and non‐stimulated neurons following acute treatment with synaptic blockers (CNQX: 25 µM, D‐APV: 5 µM) or caffeine (5 mM) One‐way ANOVA followed by Tukey's multiple comparisons test *n* = 6–8 FOVs, 2 independent differentiations; F (5, 87) = 11.64 (mean ± S.E.M; ****p < 0.0001; **p < 0.01; *p < 0.05).

We further extended *cali* with an evoked activity analysis mode designed to quantify neuronal responses to optogenetic stimulation at single‐cell and network levels (Figure 5). We optically stimulated a subregion (Figure 5A) of hiPSC‐derived neurons co‐expressing CheRiff and jRCaMP1b (Figure 2C), (or the whole FOV, Figure S5) while recording activity across the entire field of view (100 ms blue pulses 1%–10% intensity; ∼4 to 100 mW cm^−^^2^). As previously reported, continuous green excitation light did not activate CheRiff [8], ensuring that observed evoked responses were driven exclusively by blue light stimulation. To enable spatially resolved analyses, *cali* incorporates stimulation masks that automatically classify ROIs based on their position relative to the stimulated region (Figure 5A). Subregion‐specific stimulation allowed segregation of stimulated and non‐stimulated neuron populations within the same FOV, enabling direct comparison of local and network‐wide activity following stimulation. In Figure 5 we show all the denoised ΔF/F traces sorted by stimulated and non‐stimulated, with the detected calcium events/peaks overlaid.

We extracted fluorescence signals from individual ROIs, overlaid the stimulation timing obtained from the metadata to detect stimulus‐induced calcium transients (blue lines), and quantified evoked response amplitudes from normalized denoised ΔF/F traces (Figure 5B). We then used *cali* to quantify the connectivity using optogenetic stimulation by computing pairwise Pearson correlation matrices from denoised ΔF/F traces and spatially sorting the analysis (Figure 5C–E). We quantified functional correlation between neurons located within the stimulated region (orange box) and non‐stimulated neurons in the same FOV. When we computed correlations across the full recording, stimulated neurons exhibited higher median correlation values than non‐stimulated neurons (median 0.879 vs. 0.736), yielding a global median correlation of 0.775 (Figure 5C) and indicating strong overall network synchrony. When we restricted the analysis to stimulation windows (±250 ms around each stimulus), functional correlation increased markedly across the neuronal population (Figure 5D). During these epochs, both stimulated and non‐stimulated neurons showed elevated median correlation values (stimulated: 0.959; non‐stimulated: 0.905), resulting in a global median correlation of 0.923. By contrast, when we computed correlations during non‐stimulation periods while excluding ±250 ms around each stimulus, network correlation decreased relative to stimulation epochs (Figure 5E). Under these conditions, stimulated neurons maintained moderate correlation (median 0.863), whereas non‐stimulated neurons exhibited a larger reduction in correlation (median 0.665), producing a global median correlation of 0.730. Together, these results show that *cali* resolves dynamic, state‐dependent changes in functional connectivity and distinguishes baseline network structure from transient stimulation‐induced synchronization. To further demonstrate this capability, we plotted the evoked response amplitude according to the irradiance and found that stimulated neurons consistently exhibited higher response amplitudes across all irradiance levels, with saturation occurring near ∼48 mW cm^−^^2^ whereas non‐stimulated neurons showed weaker responses consistent with network propagation (Figure 5F). Finally, as a proof‐of‐concept to assess the sensitivity of the evoked activity pipeline to pharmacologically induced changes in network excitability, we modulated synaptic transmission using CNQX and D‐APV, as well as caffeine. Synaptic blockade significantly reduced calcium responses in non‐stimulated neurons compared to vehicle control, indicating suppression of network‐mediated activity. In contrast, caffeine enhanced responses in non‐stimulated neurons, reflecting increased excitability and network coupling (Figure 5G). Together, these results demonstrate that *cali* enables robust quantification of optogenetically evoked single‐cell responses while simultaneously capturing network‐level dynamics and their modulation by pharmacological perturbations.

### 2.5 Application in Disease Modeling

**FIGURE 6:**
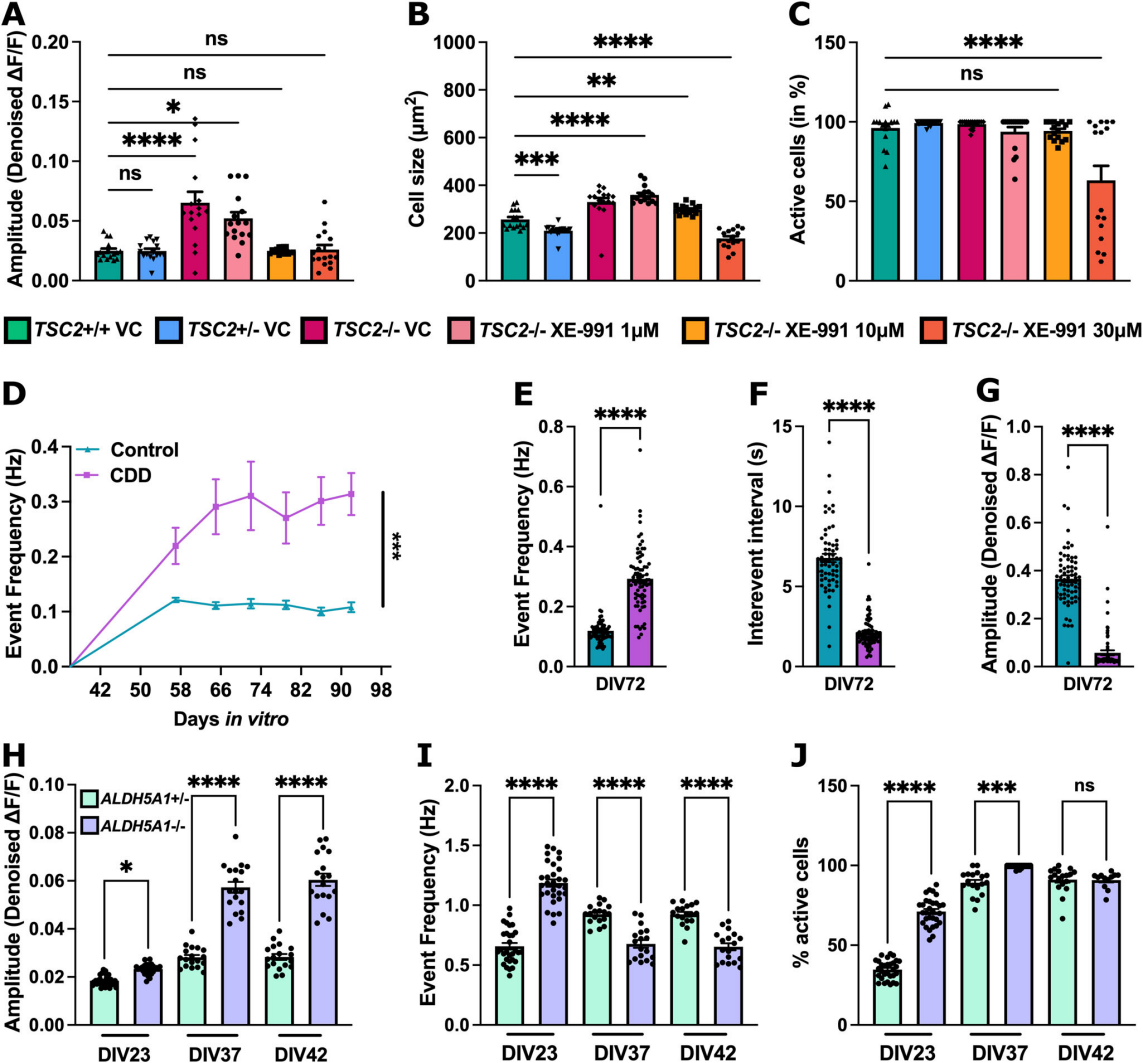
Application in disease models. (A–C) Quantification of the activity in *TSC2* ^+/+^, *TSC2* ^+/−^, and *TSC2* ^−/−^ hiPSC−derived neurons chronically treated with XE‐991 at (1, 10, and 30 µM) showing (A) increased in amplitude in *TSC2*‐deficient neurons, rescued in the XE‐991 treated conditions, (B) cell size showing significantly larger cells *TSC2*‐deficient neurons compared to controls unchanged upon treatment with XE‐991 at 1 and 10 µM but decreased at the highest concentration (30 µM) and (C) percentage of active cells, decreased in the treated condition with 30 µM. (A–C) One‐way ANOVA followed by Dunnett's multiple comparison test (A) F (29, 438) = 18.69; (B) F (29, 450) = 50.69; (C) F (29, 450) = 9.391, *n* = 3 independent differentiations, 8FOVs/condition; *TSC2* ^+/+^: 178 ROIs; *TSC2* ^+/−^: 852 ROIs; *TSC2* ^−/−^: 347 ROIs; *TSC2* ^−/−^ 1 µM XE−991: 471 ROIs; *TSC2* ^−/−^ 10 µM XE‐991: 517 ROIs; *TSC2* ^−/−^ 30 µM XE‐991: 298 ROIs. (D–G) Quantification of the activity in CDD and sex matched parental control with (D) longitudinal quantification of event frequency in hertz showing sustained network hyperexcitability in CDD neurons (E) At DIV72, detailed analysis revealed (F) significantly shorter inter‐event intervals and (G) reduced calcium event amplitudes relative to controls. (E–G) Unpaired Welch's *t*‐test (E) F(73,69) = 3.626 (F) F(63,73) = 4.910 (G) F(70,69) = 1.901, n = 3 independent differentiation, ≈70 FOVs/condition with Control: 7293 ROIs; CDD: 2917 ROIs. (H–J) Quantification of the activity in SSADH‐deficient neurons *ALDH5A1* ^−/−^ and sex matched parental control *ALDH5A1* ^+/−^ at three timepoints DIV23, DIV37 and DIV42 with (D) quantification of amplitude showing increase in *ALDH5A1* ^−/−^, (I) event frequency showing more frequent events at DIV23 and lower frequency as the network mature at DIV37 and 42 in the SSADH deficient neurons and (J) percentage of active cells increasing over time in both conditions while a significantly greater proportion of SSADH‐deficient neurons were active at early developmental stages (DIV23). (H–J) One‐way ANOVA followed by Dunnett's multiple comparison test (H) F (5, 126) = 185.4; (I) F (5, 126) = 65.27; *n* = 3 independent differentiations, 18FOVs/condition; *ALDH5A1* ^+/−^: 978 ROIs; *ALDH5A1* ^−/−^: 944 ROIs (mean ± S.E.M; ****p < 0.0001; **p < 0.01; *p < 0.05; ns = non‐significant).

To validate the robustness and versatility of our acquisition and analysis pipeline across distinct neurodevelopmental disorders, we applied it to models of TSC, CDD, and SSADHD using complementary strategies for GECI expression, indicator types, and neuronal differentiation protocols. For TSC, we generated an isogenic allelic series of hiPSC lines with stable GCaMP6s integration into the AAVS1 safe harbor locus in *TSC2* ^+/+^, *TSC2* ^+/−^, and *TSC2* ^−/−^ backgrounds (Table 1, Figure 2B, Figure 6A–C, Figure S1), ensuring consistent long‐term expression from early differentiation through neuronal maturation and chronic treatments. In parallel, for CDD (Table 1 and Figure 6D–G) and SSADHD models (Table 1 and Figure 6H–J), we transduced post‐mitotic neurons with GCaMP6s or jRCaMP1b via lentiviral delivery (Figure 2A,C,D). Neuronal differentiation employed an accelerated cortical differentiation protocol for CDKL5 and hNGN2‐mediated differentiation protocol for TSC and SSADH deficiency, enabling assessment of disease‐associated phenotypes across multiple models.

Using our automated pipeline, we recorded spontaneous calcium activity in *TSC2* ^‐/−^ neurons at DIV42 and observed a significant increase in calcium event amplitude (Figure 6A) and cell size (Figure 6B) relative to *TSC2* ^+/−^ and *TSC2* ^+/+^ controls, consistent with previously reported phenotypes in hiPSC‐derived neurons.^15^

To probe therapeutic potential, we chronically treated *TSC2*‐deficient neurons with the potassium channel modulator XE‐991 at 1, 10, and 30 µM from DIV10 to DIV42 [39]. We found that 1 µM XE‐991 had no effect on calcium event amplitude, while 10 and 30 µM treatments significantly reduced event amplitude to control levels (Figure 6A), indicating dose‐dependent normalization of neuronal hyperactivity. XE‐991 at 1 and 10 µM did not affect cell size; however, 30 µM induced significant reductions in both cell size (Figure 6B) and the proportion of active neurons (Figure 6C). These findings were further confirmed by visible neuronal deterioration in the calcium imaging recordings. These results demonstrated that targeted potassium‐channel modulation partially ameliorated hyperexcitability phenotypes in *TSC2*‐deficient neurons. Specifically, amplitude was normalized at 10 µM, whereas cell size required 30 µM to reach control levels. No single concentration rescued all measured phenotypes concurrently (Figure 6A–C).

Next, we modeled CDD, a neurogenetic disorder characterized by early‐onset seizures and disrupted network dynamics. CDKL5‐deficient hiPSCs and isogenic controls were differentiated using an accelerated cortical differentiation protocol [27]. We dissociated organoids at DIV35, co‐cultured neurons with hiPSC‐derived astrocytes, transduced with pLV‐hSyn‐GCaMP6s, and performed longitudinal calcium imaging every 6–8 days from DIV21 to DIV56. Compared to controls, CDKL5‐deficient neurons consistently displayed elevated calcium event frequency at all timepoints, indicating sustained network hyperexcitability (Figure 6D). At DIV36, detailed analysis revealed significantly shorter inter‐event intervals and reduced calcium event amplitudes relative to controls, reflecting altered neuronal firing dynamics. These findings are consistent with previously reported functional deficits in CDKL5 deficiency [40] and underscore the sensitivity of our pipeline for detecting disease‐relevant activity changes.

Finally, to model SSADHD, a neurodevelopmental disorder linked to epilepsy, we generated *ALDH5A1* ^−/−^ patient‐derived hiPSCs and sex matched parental control *ALDH5A1* ^+/−^. Neurons were differentiated using hNGN2 overexpression, co‐cultured with hiPSC‐derived astrocytes, and profiled longitudinally at DIV23, DIV37, and DIV42 (Figure 6H–J). We observed persistently elevated calcium transient amplitudes in SSADH‐deficient neurons at all single (Figure 6H). Early in development (DIV23), calcium event frequency was significantly higher than in controls, indicating initial network hyperexcitability. As networks matured, event frequency progressively declined while high‐amplitude transients persisted, indicative of emerging hypersynchronous, burst‐like activity reminiscent of epileptiform discharges (Figure 6H,I). Additionally, a significantly greater proportion of neurons were active at early developmental stages in SSADH‐deficient cultures. Collectively, these dynamic network alterations recapitulate key phenotypes previously reported in hiPSC‐derived neuronal models of SSADH deficiency [14].

Together, these results validate the broad applicability and generalizability of our platform to capture distinct, disease‐relevant neuronal phenotypes across multiple genetic neurodevelopmental disorders, enabling mechanistic insights and providing a scalable framework for high‐content functional screening and therapeutic development.

## 3 Conclusion

The integration of functional imaging with human iPSC‐derived neuronal models offers transformative opportunities to dissect the cellular and circuit‐level mechanisms underlying neurodevelopmental disorders. Here, we present a fully integrated, scalable, and open‐source platform that combines optogenetic stimulation, GECIs, multi‐well imaging, and automated single‐cell analysis to capture both spontaneous and evoked network activity. Applying this system across three distinct neurodevelopmental disorders (TSC, CDD, and SSADHD), we demonstrated its versatility for uncovering disease‐relevant phenotypes and its utility as a discovery platform for therapeutic interventions in precision medicine.

Functional calcium imaging offers a unique advantage: longitudinal, non‐invasive measurement of large neuronal populations with single‐cell resolution. Additionally, by incorporating spatially patterned optogenetic stimulation, our platform further allows the interrogation of synaptic integration and subnetwork dynamics, revealing how altered activity disrupts both intrinsic excitability and circuit‐level function [8, 9]. Although our current implementation employs one‐photon widefield stimulation for simplicity and cost‐effectiveness, this modality can activate axons or neurons outside the intended illumination region. Soma‐targeted opsins (e.g., soma‐targeted CheRiff) [41] offer a complementary strategy to confine excitability to the soma. In addition, more spatially precise approaches, such as digital micromirror device (DMD)‐based patterned illumination, could further enhance spatial targeting and minimize activation of axons in future iterations of the platform.

Despite the availability of several calcium imaging analysis platforms that provide robust tools for quantification, most face key barriers to scalability and broad applicability. Common bottlenecks include manual or semi‐automated cell segmentation workflows, which limit throughput and reproducibility, as well as restricted capabilities for integrating multiple experimental conditions or conducting longitudinal studies in vitro [22, 23, 24]. These limitations pose particular challenges for large‐scale disease modeling and drug screening using human iPSC‐derived neurons, where experiments often involve multiple timepoints, genotypes, and pharmacological conditions.

By contrast, a major advance of our platform lies in its automation and scalability. The fully integrated acquisition and analysis pipeline, incorporating real‐time deep learning‐based segmentation, automated trace extraction, and interactive data visualization via the *cali* interface, significantly reduces user bias and technical variability, enabling reproducible, large‐scale studies across multiple differentiation batches and patient lines, making it uniquely suited for scalable, single‐cell resolution studies of circuit‐level phenotypes in neurodevelopmental disorders. We demonstrated the sensitivity of this system to detect both hyperactive and hypoactive network states in response to pharmacological modulation and to reveal gene‐specific and patient‐specific functional signatures in CDD [40, 42] and SSADHD [14] models, highlighting its potential for drug screening.

Additionally, a key technical innovation of our approach is the generation of GCaMP6s knock‐in hiPSC lines via CRISPR/Cas9‐mediated targeting of the AAVS1 safe harbor locus [19], ensuring stable, homogeneous, and early expression of GECIs throughout neural differentiation. This strategy enables consistent longitudinal imaging across development, reduces variability across experiments, and facilitates direct comparisons between isogenic control lines and patient‐derived models. Using this approach, we validated previously reported network hyperexcitability phenotypes in TSC using multielectrode array (MEA) recordings and demonstrated that pharmacological inhibition of KCNQ channels with XE‐991, a compound shown to normalize hyperactivity in TSC models [39], effectively modulates network excitability in our system.

Finally, and importantly, the modular and open‐source architecture of this platform fosters rapid dissemination, adaptation, and community‐driven development. It provides a flexible framework that can be readily extended to additional disease models, imaging modalities, and perturbations. Future directions include the uses of soma‐targeted opsins, stimulation methods such as DMD‐, spatial light modulator (SLM)‐ or holographic based patterned stimulation [43] for improved spatial resolution stimulation, and incorporation of machine learning approaches for phenotype classification and functional connectivity mapping.

Together, these developments will enhance the platform's automation and capacity to identify novel subtypes, molecular targets, and biomarkers. In summary, this work addresses a critical gap in the field by enabling scalable, high‐content functional phenotyping of human neural circuits. By combining all‐optical physiology, automated analysis, and disease modeling in patient‐specific iPSC neurons, our platform lays the foundation for mechanistic insights and therapeutic discovery in complex neurological disorders.

## 4 Experimental Section/Methods

### 4.1 Generation of Human Induced Pluripotent Stem Cells (hiPSCs)

Subjects for this study were recruited through Boston Children's Hospital, and the study protocol was approved by the institutional review board (IRB‐P00008224, IRB‐ P00016119). Informed consent was obtained from all participants and/or their parents, as appropriate. Subject characteristics and reprogramming methods have been previously described [28, 44, 45] and are listed in Table 1.

### 4.2 Generation of Knock‐in GCaMP6s hiPSCs

Based on the human PPP1R12C genomic assembly GRCh38.p13 on NCBI, gRNAs were designed to target the AAVS1 area in the intron region. sgRNA: GGGGCCACTAGGGACAGGAT (TGG). The AAVS1‐Puro‐CAG‐GCaMP6s plasmid was a gift from Xiaojun Lian (Addgene plasmid # 20896; http://n2t.net/addgene:120896; RRID: Addgene_120896). Briefly, the plasmid targets the AAVS1 safe harbor locus and contains the GCaMP6s gene under the CAG promoter and the puro resistance gene with homology‐directed repair.

The GCaMP6s reporter was inserted into the AAVS1 safe locus of the isogenic hiPSC sets using CRISPR/Cas9 techniques. The plasmid was delivered via electroporation using the Invitrogen Neon transfection system (Thermo Fisher, #MPK5000). Briefly, the hiPSCs were digested by 0.75x TrypLE Select (Gibco, #12563029) in dPBS (Gibco, #14190‐144) at 37°C for 5 min. One million single cells were electroporated with 1.1ul 100 µM sgRNA, 2 µg AAVS1‐GCaMP6s plasmid, and 1.5 µL Alt‐R S.p. HiFi Cas9 Nuclease V3 (IDT, #1081061) in the Resuspension R buffer (ThermoFisher, #MPK10096) at 1200 V, 30 ms, and 1 pulse in a cuvette. The resulting cells were plated onto one well in a Cultrex‐coated 6‐well plate (ThermoFisher, #140675) in mTeSR plus with 1:10 10x cloneR2 (StemCell Technology, #100‐0691) and 1:100 3.55 mM Nu7026 (SelleckChem, #S2893) and left at room temperature for an hour before incubating at 37°C and 5% CO_2_. 24 h later, the medium was changed to fresh mTeSR plus with 10 µM Y27632 (Cayman Chemical, 10005583). Afterward, the medium was changed to mTeSR plus for every other day feeding. When the cells reached 40%–60% confluency, 0.6 µg/mL puromycin (InvivoGen, ant‐pr‐1) was added to the mTeSR plus media for selection for 3 days. The culture was then maintained in mTeSR+ with every other day feeding until it reached about 70% confluency. 4000 single cells were seeded per 10 cm dish (ThermoFisher, #150464) for colony picking into Cultrex‐coated 96‐well plates (Corning, #353072). When the visible colonies were formed, 24 clones per genotype were expanded to 24‐well plates (ThermoFisher, #142485). Specifically, ⅓ of the cell resuspension was passaged, ⅓ was purified for gDNA, and ⅓ was cryopreserved. AmpliTaq PCR on the extracted gDNA confirmed the homozygous insertion of the calcium reporter with two pairs of primers, Leftarm‐Fw and Rightarm‐Rv; Intron‐Fw2 and Puro‐Rv (Table S2). 4–5 clones per genotype with homozygous insertion were cryopreserved. One clone per genotype was further characterized for pluripotency markers, karyotypes, and Sanger sequencing for the insertion site (Table S2). After confirming the quality of the cell lines, the clones were expanded, cryopreserved, and proceeded with downstream experiments.

### 4.3 Genomic DNA AmpliTaq PCR

After the culture reached 70% confluency, the cells were rinsed with 1 mL dPBS once and pelleted by scraping in 1 mL dPBS. The cell lysate was collected into one 1.5 mL Eppendorf tube and centrifuged at 15 000 rpm for 30 s. The genomic DNA was purified using the Monarch Spin gDNA Extraction kit (New England Biolabs, #T3010L). Briefly, the cell pellets were resuspended in 100 µL cold dPBS. 3 µL Protein kinase K and 3 µL RNase A were added to the resuspension and mixed thoroughly by brief vortexing. 100 µL cell lysis buffer was then added and mixed by vortex. The mixture was incubated at 56°C in a thermal mixer with agitation at 1400 rpm. 400 µL gDNA binding buffer was added to the resuspension and mixed thoroughly by pulse‐vortexing for 10 s. The lysate and buffer mixture were transferred into a gDNA purification column pre‐inserted into a collection tube and centrifuged first at 1000 rpm for 3 min and then at 15 000 rpm for 1 min. The flow‐through and the collection tube were discarded. The column was then washed twice with 500 µL gDNA wash buffer, followed by centrifugation at 15 000 rpm for 1 min each. The flow‐through and the collection tube were discarded again. The column was inserted into a labeled, cleaned Eppendorf tube and incubated at room temperature in 100 µL RNA‐free water preheated at 60°C for 1 min before being centrifuged again at 15 000 rpm for 1 min. The concentration of nucleic acid in the final flow‐through was determined using the Tecan Spark10M multimode microplate reader.

The PCR was carried out using the AmpliTaq Gold 360 DNA Polymerase kit (ThermoFisher, #4398881). Briefly, 25 µL of AmpliTaq Gold 360 Master Mix, 5 µL of GC enhancer, 2 µL of primers (1 µL forward and 1 µL reverse), and 18 µL of gDNA and UltraPure DNase/RNase‐Free Distilled Water (Invitrogen, #10977015) were mixed for one 50 µL reaction. Between 50–200 ng gDNA was loaded, depending on the concentration, and all samples were loaded with the same weight. The PCR reaction was run on a BioRad C1000 Touch Thermal Cycler (BioRad, #1851148). To confirm the insertion, E‐Gel 2% Agarose Gels with SYBR Safe (Invitrogen, #A42135) were used for electrophoresis. PCR products were also sent for Sanger Sequencing to ensure the correct insertion sequences.

### 4.4 Mycoplasma Testing

All cellular cultures were routinely tested for mycoplasma by PCR and only negative samples were used in this study. Media supernatants (with no antibiotics) were collected, centrifuged, and resuspended in a saline buffer. Ten microliters of each sample were used for a PCR with the followings sets of primers: Myco280_CReM (5′‐ACACCATGGGAGYTGGTAAT‐3′); Myco279_CReM (5′‐CTTCWTCGACTTYCAGACCCAAGGCAT‐3′) from the Center for Regenerative Medicine: CReM—Boston University and MGSO‐5 (5′‐TGCACCATCTGTCACTCYGTTAACCTC‐3′) and GPO‐3 (5′‐ GGGAGCAAACAGGATTAGATACCCT‐3′).

### 4.5 Immunocytochemistry and Imaging

Immunocytochemistry staining was performed to confirm the pluripotency of the generated hiPSC lines. The cells were cultured on poly‐D‐lysine (Sigma‐Aldrich, P6407‐5MG) /Laminin (ThermoFisher, #23017‐015)‐coated 96‐well plate (Cellvis, #P96‐1.5P) and fixed using 4% paraformaldehyde (PFA; Polysciences, #04018‐1) in PBS when they reached ideal confluency. Briefly, an equal amount of 8% PFA was added to each well on top of the media. The plate was incubated in 4% PFA at room temperature for 20 min, followed by three rolling washes with 100 µL/well PBS. The plate was stored at 4°C if not stained immediately. The blocking buffer was made as follows: 5% Normal Goat Serum (ThermoFisher, #50197Z), 2% Bovine Serum Albumin (Gibco, #15260‐037), and 0.1% Triton‐X 100 (Sigma‐Aldrich, #T9284‐100ML) in PBS. The fixed plate was incubated in 100 µL/well blocking buffer at room temperature for an hour before 50 µL/well primary antibody diluted in blocking buffer was added (Table S3) and stored at 4°C overnight. Approximately 16 h later, the primary antibodies were removed, and the cells were washed with 100 µL/well PBS three times. The cells were then incubated with 50 µL/well secondary antibodies (Table S3) diluted in blocking buffer at room temperature for an hour in the dark. After the incubation, the antibodies were removed, and the cells were washed with 100 µL/well PBS three times in the dark. After the washes, the cells were incubated in 4 µg/ml Hoechst (Invitrogen, #H3569) diluted in UltraPure *diH*_2_ *O* for 5 min, followed by three washes with 100 µL/well *diH*_2_ *O*. The stained plates were imaged with the ImageXpress Micro XLS High‐Content Imaging System (Molecular Devices) at 20x magnification with a 6‐step z‐stack.

### 4.6 Generation of hiPSC‐Derived Neurons (hNGN2)

We used two differentiation methods in this manuscript, the NGN2 overexpression [46] with minor changes as described in one of our previous studies [14], and an accelerated cortical organoid differentiation [27]. For the NGN2 differentiation, the vectors were a gift from Kristen Brennand transduced with lentiviral vectors that encode human NGN2 (hNGN2) under the tetracycline promoter, as well as the puromycin (pLV‐TetO‐hNGN2‐Puro, addgene # 79049; http://n2t.net/addgene:79049; RRID: Addgene_79049) or neomycin (pLV‐TetO‐hNGN2‐Neo, addgene #99378; http://n2t.net/addgene:99378; RRID: Addgene_99378) resistance gene for selection. hiPSCs were grown under feeder‐free conditions, mycoplasma negative, karyotyped after transduction, and no abnormalities were detected. The hNGN2 transduced hiPSCs were dissociated into single cells using Accutase and seeded onto Geltrex‐coated plates at a density of 100 000/cm^2^. The next day, hNGN2 expression was induced using doxycycline and selected with puromycin. Growth factors BDNF (10 ng/mL, catalog #450–02; Peprotech), NT3 (10 ng/mL, catalog #450–03; Peprotech), and laminin (0.2 mg/L, catalog #23017–015; Thermo Fisher Scientific) were added in N2 medium for the first two days. Cells were then fed with BDNF (10 ng/mL), NT3 (10 ng/mL), laminin (0.2 mg/L), doxycycline (2 µg/mL), and Ara‐C (4µM, catalog #C1768; Sigma‐Aldrich) in B27 media and fed every other day until dissociation at DIV7. Cells were then dissociated with papain (catalog #LK003178; Worthington) and DNaseI (catalog #LK003172; Worthington) and replated on 96‐well plate (Cellvis, #P96‐1.5P) or 384 plates Poly‐d‐Lysine (PDL 0.5 mg/mL; catalog P6407; Sigma Aldrich) and laminin (5 µg/mL; catalog #23017–015; Life Technologies) coated plates at a density of 1300/mm^2^ in co‐culture with hiPSC‐derived astrocytes at a density of 200/mm^2^ (Astro.4 U; Ncardia).

### 4.7 Generation of 3D Accelerated Cortical Organoids and 2D Dissociated Cultures

hiPSC clones that express wild‐type CDKL5 (BCHi005‐B) and the variant form (BCHi005‐A) derived from a female patient with CDKL5 deficiency disorder [45] were first differentiated to neural stem cells (NSCs) in a monolayer format as outlined in a previous study [27]. A large stock of NSCs was established by freezing the cells in a cryopreservation medium containing 90% culture media and 10% DMSO on day 11 of differentiation [42]. NSCs from this bank were thawed, expanded until day 14 of differentiation and were aggregated at 5 million cells/well of a 6 well plate and were differentiated to cortical organoids as previously described [27]. For dissociating the organoids and plating these for calcium imaging, first, 96‐well glass‐bottom plates (Cellvis # P96‐1.5P) were coated with 25 µg/mL poly‐L‐ornithine (PLO; Sigma Aldrich #P4957) in borate buffer (Thermofisher #28341), overnight at 37°C. The PLO solution was aspirated and the wells were next coated with 5 µg/mL laminin solution (Thermofisher #A29248) in DPBS overnight at 37°C. Laminin solution was aspirated just before use. One day prior to dissociating the organoids, astrocytes (NCardia Astro.4U) were plated onto the inner 60 wells of the poly‐L‐Ornithine/laminin‐coated 96 well glass bottom plates at 6000 cells/well in astrocyte media (Sciencell #1801) with 10 µM rock inhibitor (Cayman Chemical #10005583). The outer wells were filled with sterile water to lessen evaporation of media from the wells on the edge. On day 36 of differentiation, organoids were dissociated into single cells by incubating these in Accumax (Innovative Cell Technologies # AM105.500) for approximately 2 h at room temperature followed by quenching with 10% knockout serum replacement (Invitrogen #10828028) in DMEM. The organoids were gently triturated and the cells were passed through a 40 µm cell strainer. Cells were counted and plated on astrocytes in the 96 well plates at 40 000 cells/well in phenol red‐free BrainPhys media (Stemcell Technologies # 05791) supplemented with SM1 supplement (Stemcell Technologies # 05711), GlutaMax (Thermofisher #35050061) and BDNF (Peprotech 450‐02), NGF (Peprotech #450‐01), NT3 (Peprotech #450‐03), laminin, 10 µM rock inhibitor, and CEPT (Tocris 7991). Cells were plated in half the final volume and 24 h after plating the rest of the media without rock inhibitor and CEPT was added. Half media changes were performed using a P300 Integra multichannel pipette twice a week.

### 4.8 Calcium Imaging: Spontaneous and Evoked Activity

Cultures of iNs and hiPSC‐derived astrocytes were transduced with lentiviral particles (4 h incubation) pLV‐hSyn‐jRCaMP1b at DIV 21 for hNGN2 and DIV50 for the dissociated cortical organoids and recordings weekly starting one week post transduction. Viral particles were prepared by the Viral Core at Boston Children's Hospital. Imaging was performed on a Nikon Ti2‐E epifluorescence microscope (https://tinyurl.com/mic‐all‐optical) equipped with a Nikon 20×/0.75 NA air objective and a PCO Edge 4.2 USB sCMOS camera. Samples were maintained at 37°C with 95% humidity and 5% CO_2_ using a stage‐top incubator (Okolab, model H01‐T‐UNIT‐LB‐Plus). The change in fluorescence from jRCaMP1b was recorded at 10 Hz, binning 2, under continuous green illumination at 550 nm through a Chroma filter set: excitation filter ET570/20x, dichroic mirror T585lpxr, longpass emission filter ET590lp. The change in fluorescence from GCaMP6s was recorded at 10 or 20 Hz, binning 2, under continuous blue illumination at 470 nm through a Chroma filter set: excitation filter ET470/40x, dichroic mirror T495lpxr, emission filter ET525/50m. For the evoked activity experiments, the blue illumination for the stimulation of the light‐activated ion channel CheRiff, was provided by a blue light LED at 455 nm (Thorlabs M455L3 mounted LED with LEDD1B Driver) through a Chroma filter set: excitation filter ET470/40x, dichroic mirror T505lpxr (installed on the second filter turret of the microscope). A manual lever‐actuated iris diaphragm (Thorlabs CP20S) was installed after the blue LED in a plane conjugated with the focal plane of the microscope objective. In this way, within the field of view, different sizes of the blue illumination could be selected. The minimal stimulation field possible with this configuration, measured with the 20×/NA 0.75 objective, approximately 80 µm diameter, corresponding to an illuminated area of ∼5 × 10^3^ µm^2^, typically contains on average 4 hiPSC‐derived neurons. An elliptical mirror (Thorlabs) mounted on a Thorlabs manual kinematic mount (KCB1EC/M) was placed in a plane conjugated with the back focal plane of the objective so that steering it would allow for positioning the blue illumination in different part of the FOV. The iris and kinematic mount combination, allows to control illumination size and position. This was achieved by relaying both the iris plane and the steering‐mirror plane through a set of Thorlabs lenses (L_1_ = AC254‐100‐A, L_2_ = AC254‐100‐A, and L_3_ = AC254‐150‐A) arranged in two 4f configurations (Figure 3A). First, the iris plane (IP_3_) is relayed onto the intermediate image plane (IP_2_) using lenses L_1_ and L_2_ in a 4f configuration. IP_2_ is then relayed onto the objective's image plane (IP_1_) by placing lens L_3_ one focal length away from both IP_2_ and the objective's back focal plane (BFP_1_). In parallel, the steering mirror (BFP_2_) is positioned one focal length away from lens L_2_ (between L_1_ and L_2_) and is relayed to the objective's back focal plane (BFP_1_) using lenses L_2_ and L_3_ in a 4f configuration.

### 4.9 Multi‐Dimensional Acquisition (MDA) Interface

To support scalable, open‐source data acquisition for calcium imaging and optogenetic stimulation across multi‐well formats, we developed a modular graphical user interface (GUI) built on the Python‐native pymmcore‐plus ecosystem (https://pymmcore‐plus.github.io/pymmcore‐plus/). While Micro‐Manager offers broad hardware compatibility, its Java‐based architecture limits workflow customization. Our GUI, micromanager‐gui (https://github.com/fdrgsp/micromanager‐gui), integrates pymmcore‐plus, pymmcore‐widgets, and useq‐schema to enable synchronized stimulation, real‐time segmentation, and standardized acquisition protocols within a modern, extensible Python framework. It remains fully compatible with existing Micro‐Manager configurations, requiring no hardware or settings changes.

We extended the highly functional MDAWidget from the pymmcore‐widgets library to create an intuitive interface for configuring multi‐dimensional acquisitions, tailored to the needs of calcium imaging experiments. This widget supports key imaging modes including multi‐channel, time‐lapse, Z‐stack, multi‐position, and grid acquisition, while maintaining a user‐friendly and flexible layout. Importantly, MDA settings can be easily saved and reloaded, enabling reproducibility of acquisition parameters across experiments. The widget also includes support for hardware autofocus devices, such as the Nikon Perfect Focus System (PFS) used in our experiments, which is essential for maintaining stable focus across wells during long or large‐scale imaging sessions. To facilitate imaging across multi‐well plates, we incorporated the HCSWizard feature from the MDAWidget, which provides a robust interface for configuring high‐content screening workflows. This tool allows users to select from standard plate formats (e.g., 96‐ or 384‐well) and perform precise plate calibration, with options to save, reload, and validate calibrations across sessions. It also supports flexible field‐of‐view (FOV) selection per well, offering three modes: acquisition at the well center, a reproducible random sampling of FOVs, or a defined grid layout within each well. This flexibility makes it easy to tailor acquisition strategies to different experimental needs while maintaining reproducibility and spatial consistency across datasets. For calcium imaging experiments, our preferred data format is OME‐Zarr, which offers efficient, scalable storage well‐suited to large, multidimensional datasets. The MDAWidget natively supports saving acquisitions in OME‐Zarr, with Google TensorStore integrated as the backend for data writing. This allows chunked, asynchronous saving during acquisition, enabling large time‐series datasets to be stored without requiring full in‐memory representation. By leveraging this integration, the GUI ensures fast, reliable acquisition while maintaining compatibility with modern analysis pipelines that support OME‐Zarr.

### 4.10 Data Analysis

The *cali* GUI enables rapid data visualization and analysis at the level of individual positions or entire well plates, providing a timely overview of neuronal activity. Users can create customizable plate maps to annotate genotypes and treatment conditions, allowing for intuitive comparison of calcium activity across experimental groups. Annotated single‐ROI data are automatically grouped by condition and FOV, and individual csv files summarizing each measured parameter are generated upon completion of the analysis. A more detailed description can be found at *cali* github page: https://github.com/fdrgsp/cali.

### 4.11 Quantitative Evaluation of Segmentation With a Custom Trained Cellpose Model

We quantitatively evaluated our custom‐trained Cellpose model to assess the performance improvement over the pretrained Cellpose Cyto3 model [30]. Performance was assessed on a test set of 25 images with reference instance masks. For each model, we computed Dice [32], Panoptic Quality (PQ) [33], SoftPQ [34], and mean average precision (mAP) [35]. PQ and mAP are instance‐level metrics that penalize both missed objects and spurious detections. SoftPQ is an extension of PQ that accounts for partial overlaps between predicted and reference instances, and Dice measures region overlap. To prepare the ground truth, we used the Cellpose model to generate initial masks, and then an expert evaluator performed manual curation and validation of the instance masks using Napari [46].

### 4.12 Compute ΔF/F_0_

After obtaining the segmentation file to delineate each neuronal soma and extracting the raw fluorescence traces over time for each ROI, the analysis proceeds with data pre‐processing and ΔF/F_0_ calculation. This step uses a sliding window approach combined with percentile‐based background estimation. For each timepoint, the baseline fluorescence F_0_ is defined as the 10th percentile of the fluorescence values within a user‐defined temporal window. The ΔF/F_0_ trace is then computed as the fractional change in fluorescence relative to this baseline, ΔF/F_0_ = (F − F_0_) / F_0_.

To extract the underlying neural activity from the calcium signals, we applied deconvolution to the ΔF/F_0_ traces using the OASIS algorithm [36].

### 4.13 Peak Detection

The next step in the analysis pipeline is the detection of calcium transients, which are identified as peaks in the denoised ΔF/F_0_ traces. To achieve this, we employed the scipy find_peaks function, allowing for user‐defined control over key detection parameters including prominence, minimum peak height, and minimum inter‐peak distance. The noise levels for each ΔF/F_0_ ROI traces are estimated using the OASIS package. Two modes can be used in *cali* to determine the minimum peak height: a (noise) multiplier mode, where the minimum height is computed dynamically for each ROI as a user‐defined multiple of the OASIS estimated noise level or a global mode, where a user‐defined fixed absolute value is applied to all the ROIs. The prominence threshold is always set adaptively for each ROI as the product of the OASIS estimated noise and a user‐defined multiplier. Additionally, a minimum distance constraint between peaks can be enforced to prevent multiple detections of the same event.

### 4.14 Features Extraction

Once peaks are detected, their amplitudes are extracted directly from the denoised ΔF/F_0_ trace at the corresponding time points. In addition to peak amplitude, several basic features are computed for each ROI, including event frequency, inter‐event interval (IEI), cell size (based on segmentation mask area) and percentage of active cells. Furthermore, population‐level metrics such as pairwise Pearson correlation between ROIs are also calculated, providing insights into coordinated network activity.

### 4.15 Evoked Activity Experiments

For experiments involving optogenetic stimulation, the pipeline includes a dedicated evoked activity analysis module. Regions of interest (ROIs) are classified as “stimulated” if more than 10% of their area overlaps with a user‐defined stimulation mask. Detected peaks are then classified as “stimulated” if they occur within a five‐frame window following a stimulation pulse. A binary search algorithm is used to efficiently match peaks to stimulation events. Peak amplitudes are further grouped based on stimulation parameters, such as pulse duration and LED power. The system supports various forms of LED calibration (e.g., linear, quadratic, exponential, power‐law, logarithmic), allowing for flexible power‐response analysis.

### 4.16 Visualization

At the multi‐well level, *cali* enables comparative analysis across wells grouped by genotype, treatment, or experimental condition. Summary statistics are displayed using grouped bar plots with pooled standard errors. For evoked experiments, comparative metrics between stimulated and non‐stimulated populations are also available, including power‐response relationships and stimulation‐dependent activity profiles. All visualizations dynamically update in response to changes in analysis parameters, offering an integrated and interactive environment for data interpretation. With real‐time feedback, flexible filtering, and exportable figures, the *cali* supports both exploratory analysis and publication‐quality visualization.

### 4.17 Statistical Independence and Experimental Replicates

In this study, we performed independent differentiations (biological replicates) and imaged multiple FOVs and ROIs (technical replicates) as detailed in the figure legends. Considering that the technical replicates derived from the same differentiation are not statistically independent, pooling FOV‐/ROI‐level measurements for statistical testing may underestimate the true within‐group variance. Given the limited number of independent differentiations, the statistical power at the biological‐replicate level is constrained. For future studies with larger datasets, statistical approaches that account for hierarchical data structure such as mixed‐effect models should be considered to more appropriately model nested measurements.

## Author Contributions

**Wardiya Afshar‐Saber**: Writing – original draft, Visualization, Validation, Supervision, Methodology, Investigation, Formal analysis, Data curation, Conceptualization, Project administration, Funding acquisition. **Federico M. Gasparoli**: Writing – original draft, Visualization, Validation, Supervision, Methodology, Investigation, Formal analysis, Data curation, Conceptualization. **Ziqin Yang**: Writing – review & editing, Investigation, Methodology, Validation, Data curation. **Nicole A. Teaney**: Investigation, Data curation.  **Rachel Hobson**: Investigation. **Lahin Lalani**: Investigation.  **Gayathri Srinivasan**: Investigation. **Dosh Whye**: Methodology, Resources. **Ranit Karmakar**: Methodology, Resources. **Elizabeth D. Buttermore**: Methodology, Resources. **Kellen D. Winden**: Methodology, Resources. **Cidi Chen**: Investigation, Methodology, Resources. **Mustafa Sahin**: Writing – review & editing, Supervision, Data curation, Project administration, Funding acquisition.

## Funding

This study was supported by the Rosamund Stone Zander and Hansjoerg Wyss Translational Neuroscience Center, Boston Children's Hospital Equipment and Core Resources Allocation Committee award (WAS) and Congressionally Directed Medical Research Programs W81XWH2110209 (MS).

## Conflicts of Interest

Mustafa Sahin reports grant support from Novartis, Biogen, Astellas, Aeovian, Bridgebio, and Aucta. He has served on Scientific Advisory Boards for Novartis, Roche, Regenxbio, SpringWorks Therapeutics, Jaguar Therapeutics and Alkermes.

## Supporting information




**Supporting File**: advs74649‐sup‐0001‐SuppMat.docx

**Supporting File**: advs74649‐sup‐0002‐VideoS1.mp4

**Supporting File**: advs74649‐sup‐0003‐VideoS2.mov

## Data Availability

The data that support the findings of this study are openly available in FigShare at (https://doi.org/10.6084/m9.figshare.29599163). All data reported in this paper will be shared by the lead contact by request. All code is available on github (https://github.com/fdrgsp/micromanager‐gui).
