# Supplementary material for: An Open‐Source Pipeline for Calcium Imaging and All‐Optical Physiology in Human Stem Cell‐Derived Neurons
Source: Adv Sci (Weinh). 2026 Mar 9;13(26):e15887. doi: 10.1002/advs.202515887 (PMC13159138; doi:10.1002/advs.202515887)
Supplement: Supplementary file 1 — Supporting File: advs74649‐sup‐0001‐SuppMat.docx [file ADVS-13-e15887-s001.docx]

Supporting Information

**An open-source pipeline for calcium imaging and all-optical physiology in human stem cell-derived neurons**

*Wardiya Afshar-Saber,^1,2,†,*^ Federico M. Gasparoli,^3,†^ Ziqin Yang,^1,2^ Nicole A. Teaney,^1,2^ Rachel Hobson,^1,2^ Lahin Lalani,^1,2^ Gayathri Srinivasan,^2,4^, Dosh Whye,^2,4^ Ranit Karmakar,^3^ Elizabeth D. Buttermore,^2,4^ Kellen D. Winden,^1,2^ Cidi Chen,^2,4^ Mustafa Sahin^1,2,4,**^*

^1^Department of Neurology, F.M. Kirby Neurobiology Center, Boston Children’s Hospital, Harvard Medical School, Boston, MA 02115, USA.

^2^Rosamund Stone Zander and Hansjoerg Wyss Translational Neuroscience Center, Boston MA 02115, USA.

^3^Department of Systems Biology, Harvard Medical School, Boston MA 02115, USA.

^4^Human Neuron Core, Boston Children’s Hospital, Boston MA 02115, USA.

^†^These authors contributed equally

^*^Co-corresponding author: [wardiya.afsharsaber@childrens.harvard.edu](mailto:wardiya.afsharsaber@childrens.harvard.edu)

^**^Co-corresponding author: [mustafa.sahin@childrens.harvard.edu](mailto:mustafa.sahin@childrens.harvard.edu)

**Figure S1:** Generation of the TSC2 full allelic series GCaMP6s reporter lines.

**Figure S2:** micromanager-gui

**Figure S3:** *cali*

**Figure S4:** Quantitative evaluation of segmentation with custom trained Cellpose model.

**Figure S5:** Evoked Activity Analysis Pipeline (full field of view)

**Table S1:** Mean quantitative segmentation metrics for Cellpose models.

**Table S2:** Primer sequences to confirm the plasmid insertion (AAVS1-Puro-CAG-GCaMP6s).

**Table S3:** Antibodies used for pluripotency marker staining.

**Other Supplementary Materials for this manuscript include the following:**

**Movie S1:** Modular acquisition platform for calcium imaging and all-optical physiology.

**Movie S2:** *cali.*

*
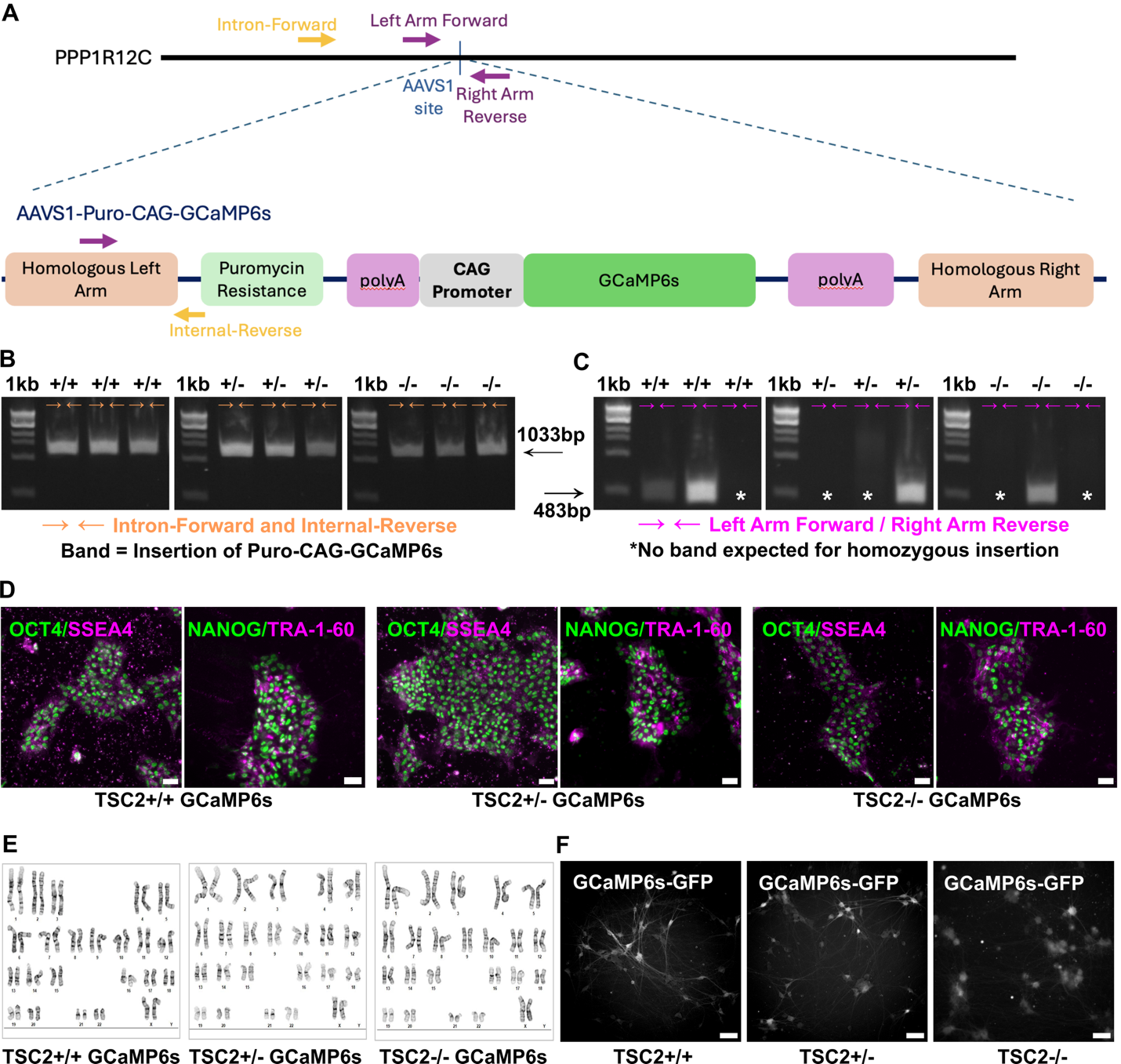
*

**Figure S1 - Generation of the TSC2 full allelic series GCaMP6s reporter lines.**

(**A**) Diagram of the strategy for the generation of the GCaMP6s reporter lines. (**B**) TSC2 full allelic series GCaMP6s reporter lines, primers: Intron-Forward and Internal-Reverse in orange on (A) and band size 1033bp. (**C**) TSC2 full allelic series GCaMP6s reporter lines, primers: Left Arm Forward and Right Arm Reverse in magenta in (A) and band size 483bp. No band expected for homozygous insertion, marked with a star (*). (**D**) Maintenance of pluripotency confirmed by expression of pluripotency markers: NANOG (Nanog homeobox x in green) and TRA-1-60 (podocalyxin in magenta), OCT4 (octamer binding transcription factor 4 in green) and SOX2 (SRY-Box Transcription Factor 2 in magenta), in undifferentiated pluripotent hiPSC colonies for the TSC2 full allelic series after insertion of GCaMP6s. (**E**) G-banded karyotype for the TSC2 full allelic series post-gene editing showing a normal karyotype for all lines (**F**) GCaMP6s-GFP expression following differentiation of the TSC2 full allelic hiPSCs series, at DIV42, scale bar 100µm.


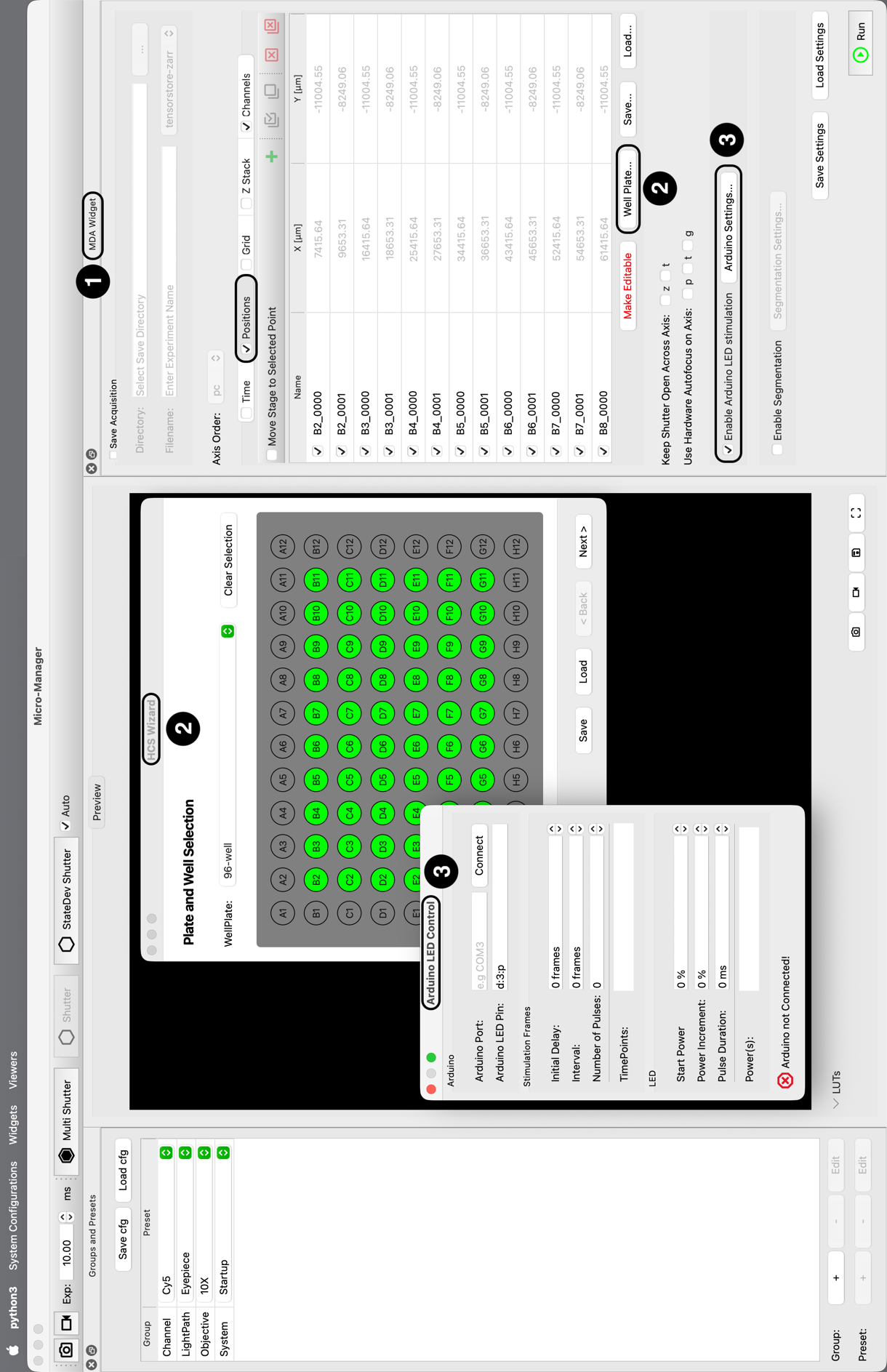


**Figure S2** – micromanager-gui user interface

The *MDAWidget* (1) to setup multi-dimensional acquisitions, the *HCSWizard* (2) to setup well plate fovs and the *Arduino Control* widget (3) for the optogenetic evoked experiments.


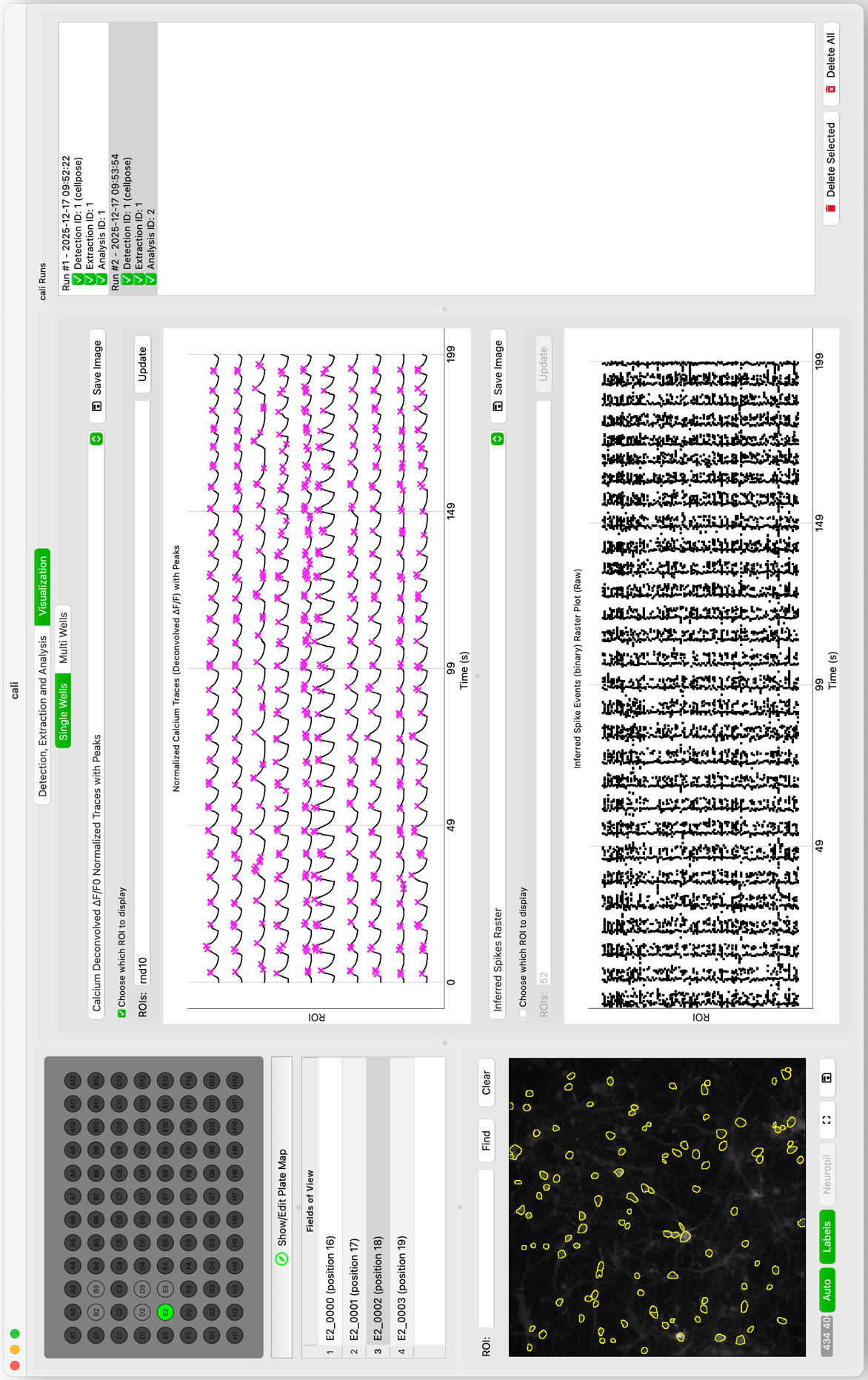


**Figure S3** – cali user interface.

A more detailed description can be found at *cali* github page: https://github.com/fdrgsp/cali


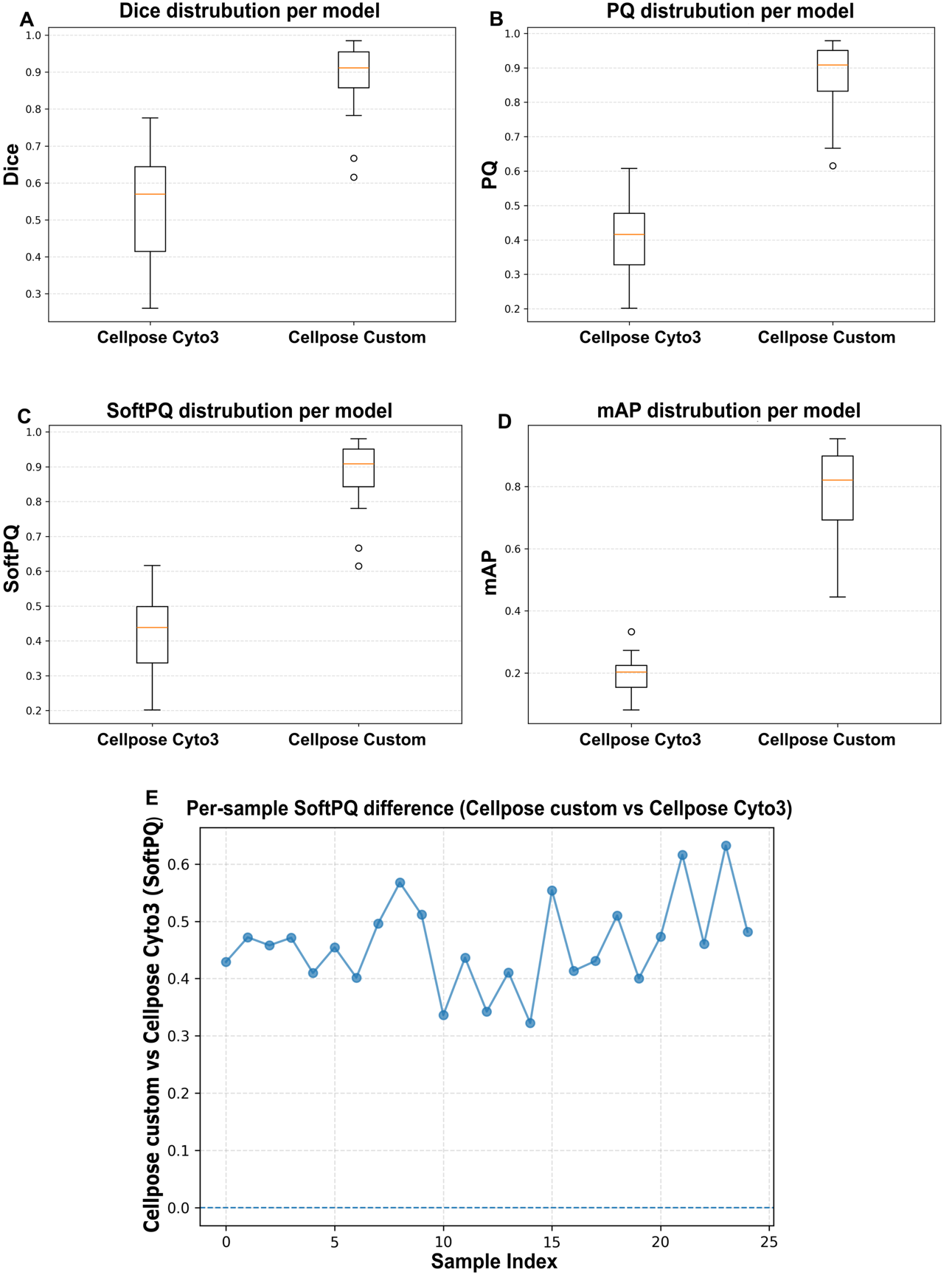


**Figure S4 - Quantitative evaluation of segmentation with custom trained Cellpose model.**

(**A-D**) Boxplots showing the distribution of Dice (A), PQ (B), SoftPQ (C), and mAP (D) scores across the 25 test images for each model. The Cellpose Custom distribution (rightmost in each panel) shows a clear upward shift compared to Cellpose Cyto3, with higher median scores (orange line) and interquartile ranges (box boundaries). (**E**) Per-sample performance difference between Cellpose Custom and Cellpose Cyto3. This line plot shows the difference in SoftPQ score (Cellpose Custom minus Cellpose Cyto3) for each of the 25 test samples (Sample index). The SoftPQ difference is positive for all samples, confirming that the fine-tuned model consistently outperformed the generalized Cellpose 3 model on every image in the test set.

~~
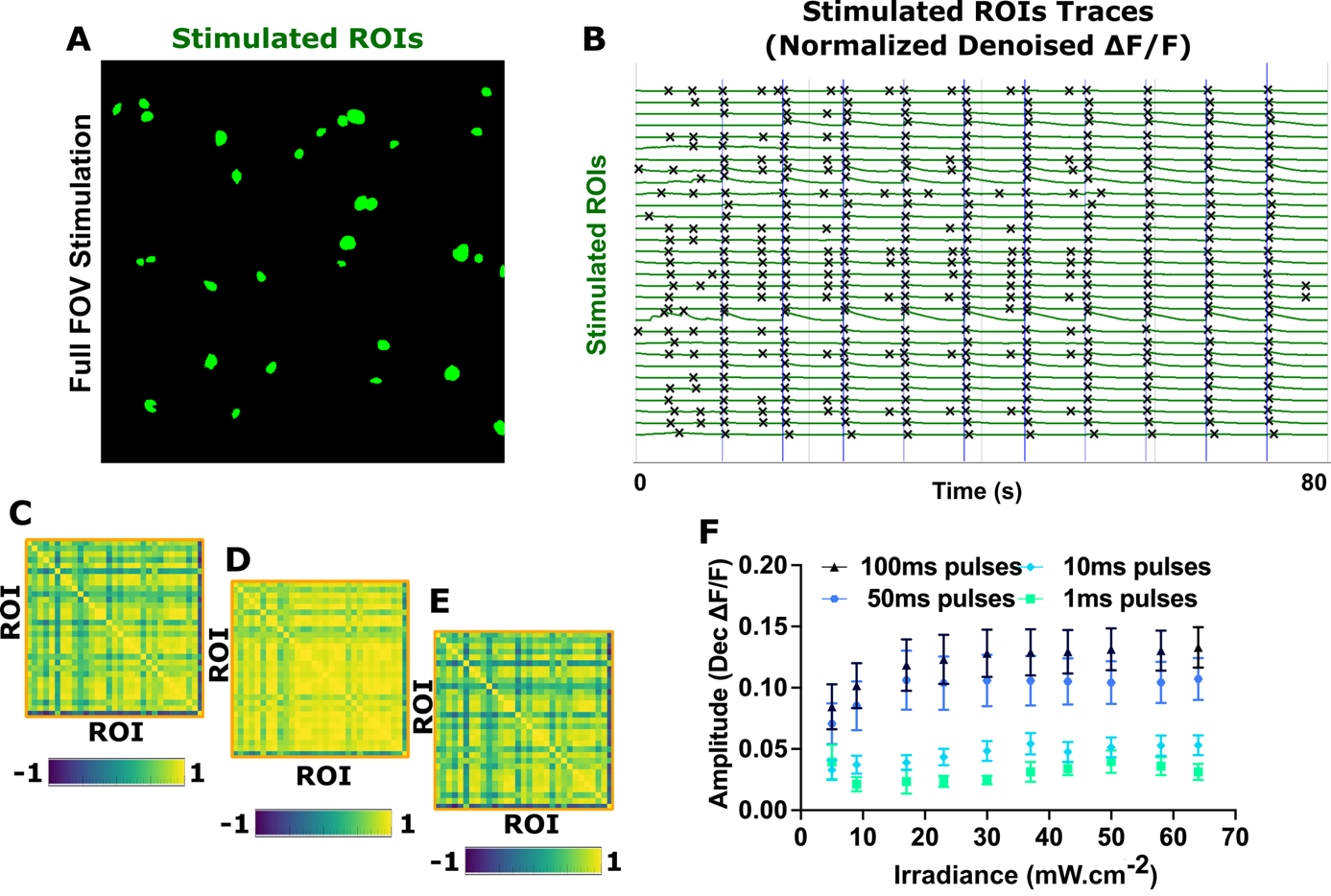
~~

**Figure S5: Evoked Activity Analysis Pipeline (full field of view)**

Neurons co-expressing CheRiff and jRCaMP1b were stimulated with 100ms blue light pulses applied in 1% increments from 1% to 10% intensity (10 pulses; ~ 4mW cm^-^² to ~ 65mW cm^-^²). (A) Segmented FOV showing stimulated ROIs in green. (B) Normalized Denoised ΔF/F from stimulated ROIs with blue lines indicating stimulation pulses. (C) Pairwise Pearson Correlation (Denoised ΔF/F) (Sorted: 31 Stim, 0 Non-Stim) | Stim median: 0.821 | Global median: 0.821 (D) Pairwise Pearson Correlation (Stim Windows ±250ms - Denoised ΔF/F) (Sorted: 31 Stim, 0 Non-Stim) | Stim median: 0.937 | Global median: 0.937. (E) Pairwise Pearson Correlation (Non-Stim Periods, excluding ±250ms - Denoised ΔF/F) (Sorted: 31 Stim, 0 Non-Stim) | Stim median: 0.806 | Global median: 0.806. (F) To further characterize stimulus-response relationships, we varied pulse duration (1, 10, 50, and 100ms). Short pulses (1ms and 10ms) elicited responses above baseline noise, while longer pulses (50ms and 100ms) produced more robust and quantifiable activity, plateauing at ~ 48 mW cm^-^².

**Table S1: Mean quantitative segmentation metrics for Cellpose models.**

Mean scores were calculated over a test set of 25 microscopy images with manually validated ground truth instance masks. The Cellpose Custom model, fine-tuned on the target dataset, achieved substantially higher performance across all four metrics: Dice (region overlap), Panoptic Quality (PQ), SoftPQ (an overlap-robust extension of PQ), and mean Average Precision (mAP) than the Cellpose Cyto3. The highest score for each metric is shown in bold.

| **Metric** | **Cellpose Cyto3** | **Cellpose custom** |
| --- | --- | --- |
| **Dice** | 0.5317 | **0.8869** |
| **PQ** | 0.4025 | **0.8776** |
| **SoftPQ** | 0.4198 | **0.8795** |
| **mAP** | 0.1932 | **0.7829** |

**Table S2: Primer sequences to confirm the plasmid insertion (AAVS1-Puro-CAG-GCaMP6s).**

| Primer name | Sequence (5’-3’) |
| --- | --- |
| Leftarm-Fw | TGC TTT CTT TGC CTG GAC AC |
| Rightarm-Rv | GGT TCT GGC AAG GAG AGA GA |
| Puro-Rv | CCG TGG GCT TGT ACT CGG TCA T |
| Intron-Fw2 | GCC AGC TCC CAT AGC TCA GTC |
| Fw1p | AGC TGC AAG AAC TCT TCC TC |
| Rv1p | AGG AGG CCT TCC ATC TGT T |
| Fw2 | GGG AGG ATT GGG AAG ACA ATA G |
| Rv1 | GGA AAG TCC CTA TTG GCG TTA C |
| Fw2p | CGC TCC GAA AGT TTC CTT |
| Rv2p | AGT AAC GCG GTC AGT CAG A |
| Fw3 | ATG GTA ATC GTG CGA GAG G |
| Rv2 | GGC ATG AAC ATG GTT AGC AGA G |
| Fw3p | CTA CCA CTA CCA GCA GAA CAC |
| Rv3p | TTG TAC AGC TCG TCC ATG CC |
| Fw4 | GGC ACA AGC TGG AGT ACA A |
| Rv3 | CCC AGC TCC TTG GTT GTT AT |
| Fw4p | ACG CTA TGT GGA TAC GCT GC |
| Rv4p | AAG CGA AAG TCC CGG AAA G |
| Fw5 | CCA CCA GCC TTG TCC TAA TA |
| Rv4 | CCT ACA GGT TGT CTT CCC AAC |
| Fw5p | ATG CCC TGG CTC ACA AAT AC |
| Rv5p | GGG CAT ATG TTG CCA AAC TC |

**Table S3: Antibodies used for pluripotency marker staining.**

| Staining | Antibody | Vendor | Catalog # | Host | Dilution |
| --- | --- | --- | --- | --- | --- |
| Primary antibody | Anti-OCT4 | Cell Signaling | 2840s | Rabbit | 1:250 |
|  | Anti-SSE4 | Invitrogen | 41-4000 | Mouse | 1:250 |
|  | Anti-Tra1-60 | Invitrogen | 41-1000 | Mouse | 1:250 |
|  | Anti-NANOG | Abcam | AB109250 | Rabbit | 1:250 |
| Secondary antibody | Anti-Rabbit Alexa 647 | Invitrogen | A21245 | Goat | 1:500 |
|  | Anti-Mouse Alexa 568 | Invitrogen | A11004 | Goat | 1:500 |

**Movie S1. Modular acquisition platform for calcium imaging and all-optical physiology.** Open-source acquisition platform incorporating four key features: (1) optogenetic stimulation control, (2) a customizable multi-dimensional acquisition (MDA) interface, (3) real-time segmentation with Cellpose, and (4) Slackbot-based remote acquisition control (Fig. 2). This platform allowed us to perform large-scale, automated imaging experiments across multi-well plates with high reproducibility and minimal manual intervention.

**Movie S2. *cali.***

Interactive graphical interface integrated into the Micro-Manager GUI, to streamline exploration and analysis of calcium imaging datasets. *cali* enables visualization, segmentation, and quantitative analysis of multi-well imaging experiments within an intuitive environment.
